# *Streptomyces rimosus*-inoculated soil exposure modulates metabolomic profiles under chronic stress

**DOI:** 10.1016/j.csbj.2025.10.030

**Published:** 2025-10-17

**Authors:** Sowon Yang, Jin Hee Kim, Sin-Ae Park, Myung Sook Oh, Choong Hwan Lee

**Affiliations:** a Department of Bioscience and Biotechnology, Konkuk University, Seoul 05029, Republic of Korea; b Department of Biomedical and Pharmaceutical Sciences, Graduate School, Kyung Hee University, Seoul 02447, Republic of Korea; c Department of Bio & Healing Convergence, Konkuk University, Seoul 05029, Republic of Korea; d Digital Humanities Agro–Healing Convergence Research Center, Konkuk University, Seoul 05029, Republic of Korea; e Department of Oriental Pharmaceutical Science, College of Pharmacy, Kyung Hee University, Seoul 02447, Republic of Korea; Institute of Integrated Pharmaceutical Sciences, College of Pharmacy, Kyung Hee University, Seoul 02447, Republic of Korea

**Keywords:** Chronic restraint stress, Anxiety, Soil microorganism, Metabolite profiling

## Abstract

Exposure to natural environments, including soil and microorganisms, is known to enhance emotional well-being and mitigate psychiatric disorders such as anxiety and depression. This study investigated the effects of short-term exposure to *Streptomyces rimosus*-inoculated soil, on mice with chronic restraint stress (CRS)-induced anxiety and the associated metabolic changes. Behavioral tests, including the light/dark box, open field, and novelty-suppressed feeding tests, revealed that exposure significantly alleviated anxiety-like behaviors, stress-induced neuroinflammation and hypothalamic-pituitary-adrenal (HPA) axis dysregulation of CRS mice. Untargeted metabolomics revealed alterations in amino acid metabolic pathways in the hippocampus and plasma. Potential biomarkers were highlighted, including glutamate and sphingosine in the hippocampus, and 5-hydroxytryptophan, valine, and methionine in the plasma, which showed improved metabolite levels and significant correlations with behavioral improvements, neuroinflammation, and HPA axis factors. This study provides evidence that short-term exposure to *S. rimosus*-inoculated soil may alleviate anxiety-like behaviors in a CRS mouse model by modulating amino acid metabolism and restoring potential anxiety-related biomarkers in hippocampus and plasma. These findings highlight a potential link between soil microbial exposure and stress-related physiological and metabolic responses.

## Highlights

• *S. rimosus*-inoculated soil exposure alleviated anxiety-like behaviors and improved neuroinflammation and HPA axis regulation.
• Untargeted metabolomics revealed modulation of amino acid metabolic pathways in hippocampus and plasma.
• Glutamate and sphingosine in the hippocampus were identified as potential biomarkers.
• In plasma, 5-hydroxytryptophan, valine, and methionine were recognized as potential biomarkers.

## Graphical Abstract

**Figure:**
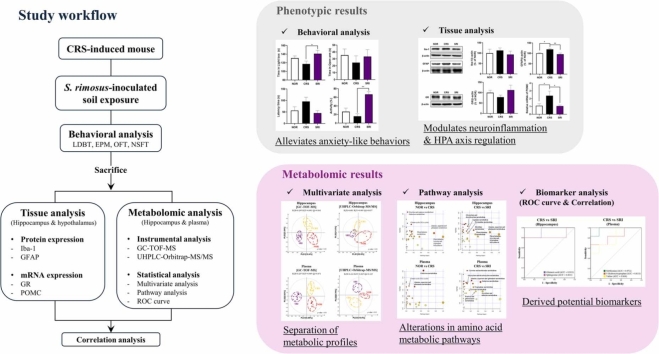

## 1 Introduction

Rapid urbanization and the rising prevalence of mental health disorders have become major public health concerns in the 21st century. Urbanization is closely associated with behavioral changes that contribute to non-communicable diseases (NCDs), including reduced physical activity, sleep disturbances, and unhealthy dietary habits [1], [2], [3], [4], [5], [6]. Among NCDs, mental disorders, particularly depression and anxiety, have become global epidemics [7], [8].

One contributing factor may be decreased exposure to natural environments, which are known to offer diverse health and wellbeing benefits [9], [10], [11], [12]. To counteract the negative effects of urban lifestyles, strategies such as the development of green spaces and nature-based interventions are being explored [13], [14].

Fragrance compounds, which are typically low-molecular-weight volatile organic compounds, can cross the blood–brain barrier and affect neurological functions [15], [16]. Upon inhalation, these molecules binds and activates to olfactory receptors in the nasal cavity. This activation generates electrical signals transmitted to the olfactory bulb and relayed to the brain, which regulates emotional and behavioral responses [17], [18].

Soil is a well-known reservoir of diverse microorganisms, and its characteristic earthy aroma originates from a complex blend of volatile organic compounds [19], [20]. *Streptomyces* species are filamentous gram-positive bacteria that emit a variety of volatile terpenoids as metabolic byproducts [21], [22]. Notably, *Streptomyces rimosus* produces high levels of geosmin and 2-methylisoborneol (2-MIB), which are largely responsible for the distinct earthy and musty odor of soil [23], [24], [25].

In human studies, exposure to soil containing *S. rimosus* has been associated with mood enhancement, reduced stress levels, and a substantial decrease in serum C-reactive protein (CRP), a known biomarker of depression [25]. A subsequent study also demonstrated that the inhalation of *S. rimosus* volatiles induced psychophysiological relaxation and frontal-lobe stress reduction in individuals with depression, possibly by modulating energy metabolism [26].

Animal models such as chronic restraint stress (CRS) have been widely used to induce anxiety- and depression-like behaviors, mimicking the physiological and behavioral characteristics of chronic psychological stress [27].

Therefore, the present study aimed to investigate the anxiolytic effects of short-term exposure to soil inoculated with *S. rimosus* KACC 20082 in a mouse model of CRS-induced anxiety. Furthermore, anxiety-related behavioral changes, factors, and metabolic alterations were examined using behavioral tests, tissue analyses, and untargeted metabolomic profiling.

## 2 Methods

### 2.1 Materials

Skimmed milk was purchased from BD Transduction Laboratories (Franklin Lakes, NJ). Protein assay reagent, acrylamide, and enhanced chemiluminescence reagent were purchased from Bio-Rad Laboratories (Hercules, CA, USA). Radioimmunoprecipitation assay (RIPA) buffer and a protease/phosphatase inhibitor cocktail were purchased from Thermo Fisher Scientific (Waltham, MA, USA). The rabbit anti-ionized calcium-binding adapter molecule-1 (Iba-1) was purchased from Millipore Biosciences (Bedford, MA, USA). Mouse β-actin antibody, mouse glucocorticoid receptor antibody and goat anti-Glial fibrillary acidic protein (GFAP) were purchased from Santa Cruz Biotechnology (Temecula, CA, USA). HPLC-grade water, methanol and 2-chloro-L-phenylalanine were obtained from Thermo Fisher Scientific (Waltham, MA, USA). Tryptic soy broth and agar were purchased from BD Biosciences (Franklin Lakes, Dickinson and company, USA). Formic acid, reagent grade methoxyamine hydrochloride, pyridine, and N-methyl-N-(trimethylsilyl)trifluoroacetamide were purchased from Sigma-Aldrich (St. Louis, MO, USA).

#### 2.1.1 Experimental overview

Six-week-old C56BL/6 male mice were purchased from Daehan Biolink Co., Ltd. (Eumseong, Korea). The mice were randomly assigned to one of three groups, with six mice per group: (1) normal group, NOR; (2) chronic restraint stress group, CRS; and (3) chronic restraint stress with exposure to soil inoculated with *S. rimosus*, SRI. The CRS and SRI groups were subjected to CRS for 6 h per day for 14 d using a restraint device made of transparent plastic (4 × 4 × 10.5 cm). From day 15, the SRI group was exposed to *S. rimosus*-inoculated soil for 1 h prior to each behavioral test for four consecutive days, and an additional 1 h exposure was performed immediately before sacrifice for tissue collection, resulting in a total of five sessions. Exposure was conducted in individual cages, with each mouse housed separately during the exposure period (Supplementary Figure S1).

#### 2.1.2 Soil mixture preparation

*Streptomyces rimosus* KACC 20082 (Korean Agricultural Culture Collection, Republic of Korea) was cultured in tryptic soy broth at 27 °C with shaking (250 rpm) for four days. The soil mixture consisted of peat moss (800 mL), perlite (320 mL), and water (80 mL). Both peatmoss and perlite were autoclaved at 121 °C for 15 min for sterilization. For the SRI group, a *S. rimosus* bacterial suspension (20 mL) was inoculated into the soil mixture. After mouse exposure, the soil mixture comprised peat moss (130 mL), perlite (55 mL), and water (13.3 mL) with additional bacterial culture medium (3.3 mL) for the SRI group only. Detailed group information and soil mixture compositions are listed in Table 1.

**Table 1:** Group information and treatments.

**No.**	**Group**	**Treatment**	**N**	**Bacteria information**
1	NOR	-	6	-
2	CRS	CRS	6	-
3	SRI	CRS + soil + bacteria	6	*S. rimosus* KACC 20082

#### 2.1.3 Behavioral tests

##### 2.1.3.1 Light-dark box (LDB) test

Each mouse was placed in an apparatus consisting of a white brightly lit compartment (14 × 12 × 12 cm; ∼600 lux) and a black dark compartment (14 × 12 × 12 cm; <30 lux). Mice were initially placed in the light compartment facing the dark compartment, and their exploratory behavior was recorded for 5 min. The time spent in the light box was recorded to assess anxiety-like behavior.

##### 2.1.3.2 Elevated plus maze (EPM) test

The EPM test was conducted using an apparatus consisting of a central platform connected to two open arms and two closed arms (40 × 7 cm each, closed arms enclosed by 15 cm-high walls) elevated 60 cm above the floor. Light intensity was approximately 300 lux in the open arms, 250 lux in the center, and 50 lux in the closed arms. The mice were placed on the central platform at the start of the test and allowed to explore freely for 5 min. The time spent in the open arms was recorded to assess anxiety-like behavior.

### 2.2 Novelty-suppressed feeding test (NSFT)

Prior to testing, the mice were food deprived for 24 h with free access to water. During the test, a food pellet was placed at the center of a box (60 × 45 × 20 cm), and each mouse was positioned in a corner of the apparatus. The latency to approach and begin eating food was recorded for up to 5 min. Once feeding was initiated or the time limit was reached, the mouse was immediately returned to its home cage.

### 2.3 Open field test (OFT)

During the OFT, mice were individually placed in a square arena (45 × 45 × 45 cm) under evenly diffused light (∼150 lux), and their movements were tracked for 20 min using an automated video-tracking system (Biobserve, Bonn, Germany). Locomotor activity was expressed as Activity (%), which was defined as the percentage of time during which the animal was in motion relative to the total test duration. The tests were conducted under evenly diffused light.

### 2.4 Preparation of tissues

Following behavioral testing, mice were euthanized using a mixture of ketamine and xylazine in saline as an anesthetic. The hippocampus and hypothalamus were dissected for western blot and reverse transcription polymerase chain reaction (RT-PCR) analyses. The collected tissues were immediately transferred to tubes and stored at −80 °C until further use.

### 2.5 Western blot

Brain tissue was homogenized and lysed in RIPA buffer supplemented with protease and phosphatase inhibitors. The resulting lysates were denatured, separated using sodium dodecyl sulfate-polyacrylamide gel electrophoresis, and transferred onto polyvinylidene difluoride membranes. To block nonspecific binding, the membranes were incubated with 5 % skim milk for at least 1 h, followed by overnight incubation with primary antibodies at a 1:1000 dilution. After washing, the membranes were incubated with secondary antibodies (1:3000 dilution) for 1 h at room temperature. Immunoreactive bands were visualized using an enhanced chemiluminescence reagent, and band intensities were captured using Image Lab software (Bio-Rad, Hercules, CA, USA) and quantified using ImageJ software.

### 2.6 RNA extraction and RT-PCR

The mRNA expression levels were analyzed using RT-PCR. Total RNA was extracted using TRIzol reagent according to the manufacturer’s instructions. The isolated mRNA was reverse transcribed into complementary DNA (cDNA) using TOPscript™ RT DryMIX (Enzynomics, Daejeon, Republic of Korea). Quantitative RT-PCR (qRT-PCR) was performed using TOPreal™ qPCR 2X PreMIX (Enzynomics, Daejeon, Republic of Korea) on a CFX Connect Real-Time PCR System (Bio-Rad Laboratories, CA, USA). Primers were synthesized by COSMO Genetech (Seoul, Republic of Korea) with the following sequences: Pro-opiomelanocortin (POMC) forward, 5’-GAG GCC ACT GAA CAT CTT TGT C-3’ reverse, 5’-GCA GAG GCA AAC AAG ATT GG-3’; GAPDH forward, 5’-TGA ATA CGG CTA CAG CAA CA-3’ reverse, 5’-AGG CCC CTC CTG TTA TTA TG-3.’

### 2.7 Metabolite extraction from hippocampus and plasma samples

Hippocampus tissue (20 mg) was mixed with 500 µL of cold 50 % methanol containing 2-chloro-L-phenylalanine (10 mg/L) as an internal standard. The mixture was vortexed (1 min), sonicated (10 min), and homogenized (10 min at 30 Hz) using zirconium beads in a mixer mill (MM400; Retsch, Haan, Germany). After incubation at 4 °C for 60 min, the mixture was centrifuged at 13,000 rpm at 4 °C for 15 min. The supernatant was filtered through a 0.2 µm polytetrafluoroethylene (PTFE) filter and dried using a speed vacuum concentrator.

Plasma samples (200 µL) were mixed with cold methanol (800 µL) containing an internal standard. The mixture was vortexed (1 min), sonicated (10 min), and homogenized (10 min at 30 Hz). Following incubation at 4 °C for 15 min, the supernatant was filtered through a 0.2 µm PTFE filter and dried using a speed vacuum concentrator.

### 2.8 Instrumental analysis

The dried extracts were reconstituted at different concentrations for each analysis method: 1 mg/mL for UHPLC-Orbitrap-MS/MS analysis and 5 mg/mL for GC-TOF-MS analysis, with subsequent drying and derivatization. Hippocampal samples were dissolved in 50 % methanol and plasma samples were dissolved in 100 % methanol. All samples were filtered through 0.2 µm PTFE filters prior to analysis. In LC-MS, chromatographic separation was achieved using an ACQUITY UPLC HSS T3 column (100 mm × 2.1 mm, 1.8 μm particle size; Waters Co., MA, USA). LC was conducted using a Vanquish Core UHPLC system (Thermo Fisher Scientific), and mass spectra were acquired using an Orbitrap Exploris 120 Mass Spectrometer (Thermo Fisher Scientific). The GC-MS analysis was conducted using a Pegasus BT TOF mass spectrometer (LECO, ST. Joseph, MI) equipped with an RTX 5MS column (30 m × 0.25 mm, 0.25 μm particle size; Restek Corp., Bellefonte, PA). Plasma samples were injected at a 10:0 split ratio, and hippocampal samples were analyzed in splitless mode. The GC-MS derivatization procedure and instrumental parameters for the instruments were based on previously described methods by [28].

### 2.9 Data processing and statistical analysis

Statistical analyses for behavioral and western blot data were performed using GraphPad Prism 8.0. analysis of variance (ANOVA) was conducted under the standard assumptions of normality and homogeneity of variance. While additional formal tests for normality and homoscedasticity were not performed, visual inspection of the data distribution and variance patterns did not reveal major violations. Raw data files from GC-TOF-MS were converted to netCDF (*. cdf) format using the LECO Chroma TOF software (version 4.44, LECO Corp., St. Joseph, MI, USA). Peak detection, retention time correction, and peak alignment were performed using MetAlign software (RIKILT Institute of Food Safety, Wageningen, Netherlands), and the aligned data were exported to Microsoft Excel for further analysis. Raw data files from UHPLC-Orbitrap-MS/MS were converted to mzXML format using Thermo Xcalibur software (version 2.1, Thermo Fisher Scientific). The mzXML files were processed using the XCMS online software (version 3.7.1) for retention time correction, peak detection, and alignment. The aligned data were exported to Microsoft Excel for further analysis.

Multivariate statistical analyses were conducted using the SIMCA-P + ver. 12.0 software (Umetrics). PLS-DA was used to evaluate differences in metabolites between groups. The significance of the PLS-DA model was assessed using an ANOVA of cross-validated predictive residuals in the SIMCA-P + program. The metabolites were selected based on their variable importance in the projection (VIP) score of the PLS-DA model. Metabolite identification was performed by comparing the retention times and mass fragment data with reference databases, including the Human Metabolome Database (2023), National Institute of Standards and Technology database (version 2.0, 2011; FairCom, Gaithersburg, MD, USA), Wiley 9 database (Wiley VCH, Weinheim, Germany), MassBank of North America (http://massbank.us/), and an in-house library of standard compounds. Altered metabolic pathways were identified using MetaboAnalyst 6.0 (https://www.metaboanalyst.ca/) with the Kyoto Encyclopedia of Genes and Genomes database (https://www.genome.jp/kegg/pathway.html) as a reference. Pathways were considered significant when the pathway impact was > 0.1 and *p* < 0.05. Pearson correlation maps and receiver operating characteristic (ROC) curves were generated using the PASW software (SPSS Inc., Chicago, IL, USA) to examine the correlations between metabolites and phenotypic data. ROC curve analysis was performed using CRS as the reference group and SRI as the target group. Metabolites with an AUC > 0.8 and showing a significant (*p* < 0.05) correlation with the phenotypic data were proposed as biomarkers. Box-and-whisker plots were generated using Statistica 7.0 software (StatSoft France, Maisons Alfort, France). The datasets underwent log transformation of the peak intensity, and statistical differences between groups were assessed using an independent *t*-test, confirming statistical significance at *p* < 0.05.

## 3 Results

### 3.1 Exposure to *S. rimosus*-inoculated soil alleviates anxiety-like behaviors in the CRS-induced mice

**Fig. 1:**
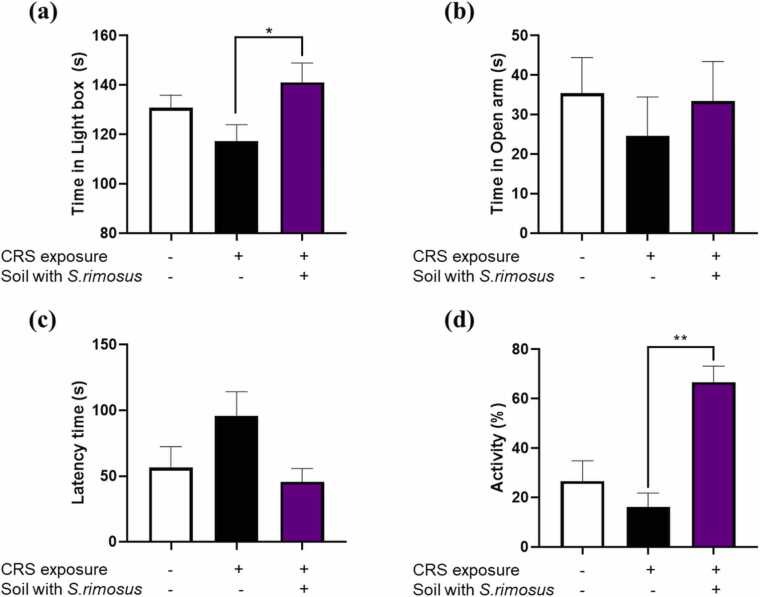
Effects of *S. rimosus*-inoculated soil on CRS-induced anxiety-like behavior in mice. (a) In the LDB test, time spent in the light box was measured for 5 min. (b) For the EPM, the time spent in the open arms was measured for 5 min. (c) Latency to initiate eating was recorded using the NSFT. (d) In the OFT, the locomotor activity was monitored over a 20 min period. Data were analyzed using a one-way ANOVA, followed by Dunnett’s multiple comparison test (* *p* < 0.05, ** *p* < 0.01).

To evaluate the effects of exposure to soil inoculated with *S. rimosus* on CRS-induced anxiety-like behaviors, the LDB, EPM, NSFT, and OFT were performed. In the LDB test, CRS exposure reduced the time spent in the light box, whereas exposure to *S. rimosus*-inoculated soil significantly increased the duration (Fig. 1a). Similarly, in the EPM test, the CRS reduced the time spent in the open arms, whereas exposure to *S. rimosus*-inoculated soil significantly restored it (Figure1b). In the NSFT, CRS increased the latency to begin eating, which was effectively reduced by exposure to *S. rimosus*-inoculated soil (Fig. 1c). In the OFT, the mice exposed to CRS showed a trend toward reduced locomotor activity. However, exposure to *S. rimosus*-inoculated soil significantly increased the activity levels compared to the CRS group (Fig. 1d).

### 3.2 Exposure to *S. rimosus*-inoculated soil modulates neuroinflammation and HPA axis regulation in the brain of CRS-induced mice

**Fig. 2:**
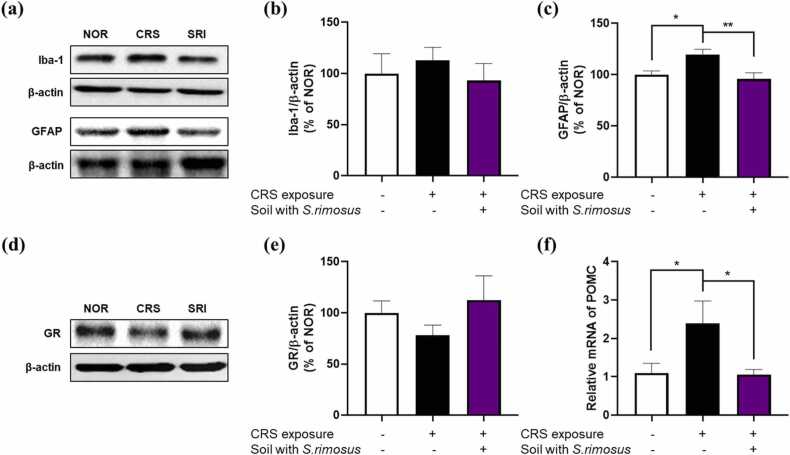
Effects of *S. rimosus*-inoculated soil on neuroinflammation and stress-related neuroendocrine responses in the CRS-induced mice brain. (a) Representative band images of Iba-1 and GFAP in the hippocampus. The protein levels of (b) Iba-1 and (c) GFAP were measured by western blotting. (d) Representative band images of GR in the hypothalamus. Protein levels of (e) GR were measured by western blotting. (f) POMC mRNA expression in the hypothalamus was measured by qRT-PCR. Data were analyzed using a one-way ANOVA, followed by Dunnett’s multiple comparison test (* *p* < 0.05, ** *p* < 0.01).

To evaluate the effects of *S. rimosus*-inoculated soil on CRS-induced neuroinflammation, we analyzed the protein expression levels of Iba-1 and GFAP in the hippocampus. Iba-1, a marker of microglial activation, showed an increasing trend in response to CRS exposure and a decreasing trend following exposure to *S. rimosus*-inoculated soil, although neither reached statistical significance. In contrast, GFAP, an astrocytic reactivity marker, was significantly elevated in CRS-exposed mice and was significantly reduced by exposure to *S. rimosus*-inoculated soil (Fig. 2a-c).

To assess the potential modulation of the hypothalamic-pituitary-adrenal (HPA) axis in response to stress, we examined GR protein levels and POMC mRNA expression in the hypothalamus. GR is a key regulator of glucocorticoid signaling and plays a crucial role in stress adaptation, whereas POMC encodes a precursor protein involved in the production of stress-related peptides, such as adrenocorticotropic hormone. CRS tended to decrease the GR protein levels, suggesting impaired glucocorticoid feedback regulation, whereas exposure to *S. rimosus*-inoculated soil restored these levels (Fig. 2d, e). Conversely, POMC mRNA expression was upregulated by CRS, reflecting overactivation of the HPA axis, but was significantly reduced following exposure to *S. rimosus*-inoculated soil, indicating the potential normalization of stress responses (Fig. 2f).

### 3.3 Untargeted metabolite profiling of hippocampus and plasma

Untargeted metabolomic profiling was performed using GC-TOF-MS and UHPLC-Orbitrap-MS/MS analyses to investigate metabolomic alterations in the hippocampus and plasma samples of the NOR, CRS, and SRI groups. Partial least squares discriminant analysis (PLS-DA) score plots revealed distinct patterns among the three groups.

**Fig. 3:**
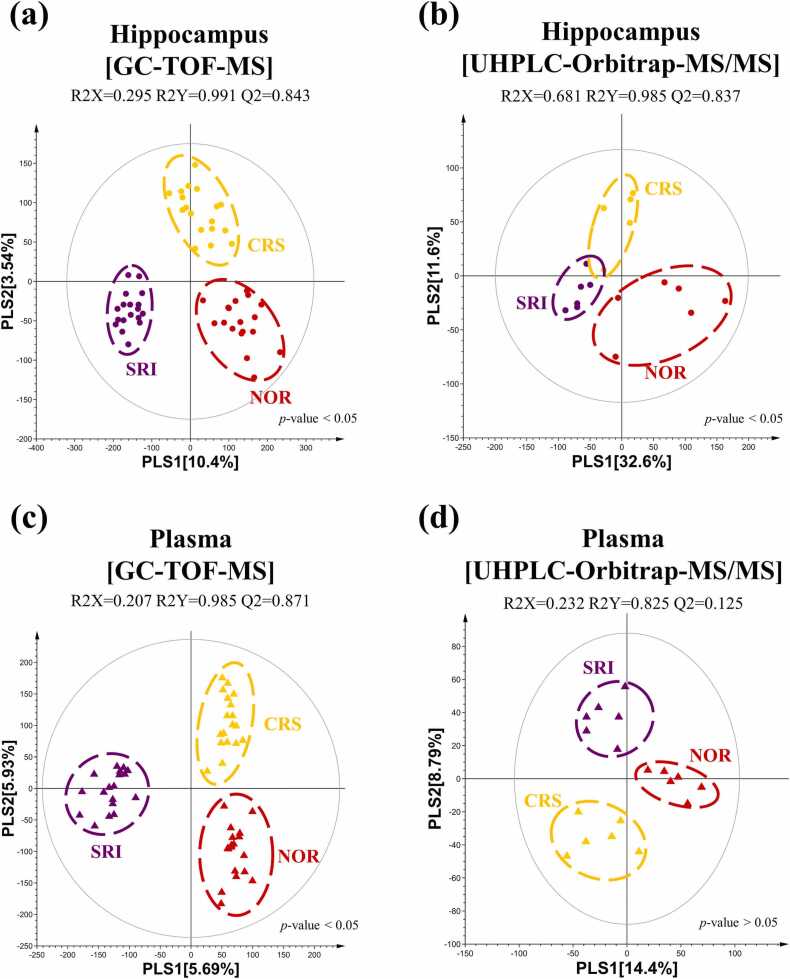
PLS-DA score plots of metabolic profiles in the hippocampus and plasma samples among NOR, CRS, and SRI groups. (a) Hippocampal metabolites analyzed by GC-TOF-MS, (b) hippocampal metabolites analyzed by UHPLC-Orbitrap-MS/MS, (c) plasma metabolites analyzed by GC-TOF-MS, and (d) plasma metabolites analyzed by GC-TOF-MS. NOR (), CRS (), SRI ().

GC-TOF-MS analysis of hippocampal samples showed clear separation among the groups with high model quality (R2X = 0.295, R2Y = 0.991, Q2 = 0.843). Similarly, UHPLC-Orbitrap-MS/MS analysis revealed distinct clustering (R2X = 0.681, R2Y = 0.985, Q2 = 0.837). Plasma metabolite analysis using GC-MS (R2X = 0.207, R2Y = 0.985, Q2 = 0.871) and LC-MS (R2X = 0.232, R2Y = 0.825, Q2 = 0.125) demonstrated clustering between the groups. Meanwhile, the plasma LC-MS dataset showed a low Q² value (0.125), indicating limited predictive performance. In the hippocampal datasets, PLS-DA score plots showed significant group separation (*p* < 0.05) in both GC (Fig. 3a) and LC-MS (Fig. 3b) analyses. Similarly, the plasma dataset exhibited grouping patterns for GC (*p* < 0.05) (Fig. 3c) and LC-MS (*p* > 0.05) (Fig. 3d). PLS-DA plots for both metrics revealed comparable metabolic profiles.

Discriminant metabolites were identified based on PLS-DA plots. In the hippocampus, 83 metabolites were identified in the GC-MS (Supplementary Table 1) and LC-MS (Supplementary Table 2), including 4 carbohydrates and derivatives, 23 amino acids and derivatives, 3 carboxylic acids, 8 fatty acids and derivatives, 20 lipids and derivatives, 10 nucleosides and derivatives, 15 others, and 25 non-identified metabolites. In the plasma, 92 metabolites were identified in GC (Supplementary Table 3) and LC (Supplementary Table 4), including 8 carbohydrates and derivatives, 13 amino acids and derivatives, 7 carboxylic acids and derivatives, 15 fatty acids and derivatives, 26 lipids and derivatives, 3 indoles and derivatives, 3 bile acids and derivatives, 3 carnitines and derivatives, 14 others, and 21 non-identified metabolites. Heatmap analysis of differential metabolites was used to visualize the metabolite levels in the hippocampus (Supplementary Figure S2a) and plasma (Supplementary Figure S2b) following CRS and SRI treatments.

### 3.4 Metabolic pathway analysis

**Fig. 4:**
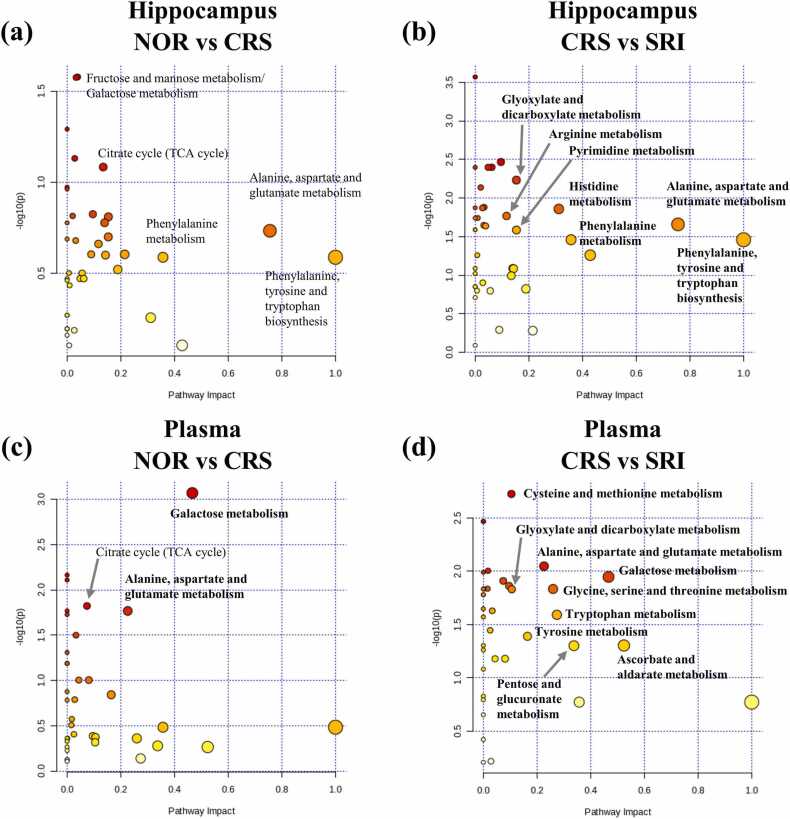
Pathway analysis of the differential metabolites in hippocampus and plasma identified by GC-MS and LC-MS, based on KEGG pathway networks. (a) Hippocampus, NOR vs. CRS; (b) Hippocampus, CRS vs. SRI; (c) Plasma, NOR vs. CRS; (d) Plasma, CRS vs. SRI. Significant pathways are shown in bold.

Metabolic pathway analyses were conducted for differential metabolites. In the hippocampus, pathways were altered in the CRS group compared to those in the NOR group (Fig. 4a), and those altered in the SRI group compared to those in the CRS group (Fig. 4b). The pathways commonly influenced by both CRS and *S. rimosus* in the hippocampus include alanine, aspartate, and glutamate metabolism; phenylalanine metabolism; and phenylalanine, tyrosine, and tryptophan biosynthesis. In the plasma, pathways altered in the CRS group compared to those in the NOR group (Fig. 4c) and those altered in the SRI group compared to those in the CRS group (Fig. 4d) were identified. Pathways commonly affected by both CRS and *S. rimosus* in the plasma included galactose metabolism; alanine, aspartate, and glutamate metabolism; and phenylalanine, tyrosine, and tryptophan biosynthesis. Among the key pathways, alanine, aspartate, and glutamate metabolism were affected by both the CRS and *S. rimosus* treatments.

### 3.5 Biomarker identification

**Fig. 5:**
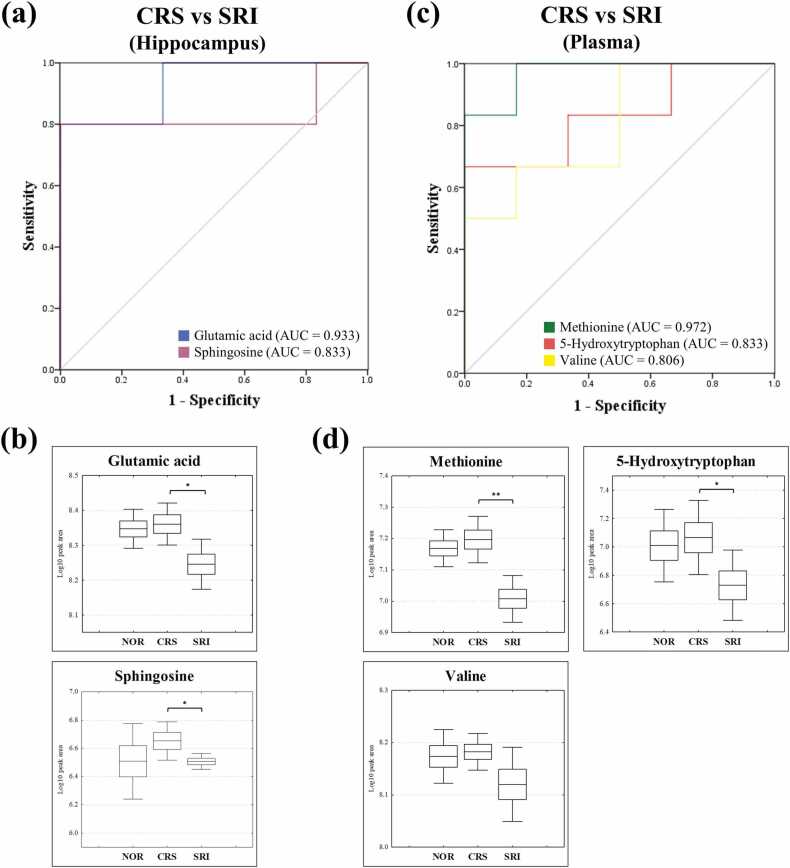
Identification of biomarkers based on ROC curves and metabolite levels of biomarkers in the hippocampus and plasma. (a) ROC curve analysis in hippocampus biomarkers; (b) Box-and-whisker plots of hippocampus biomarkers; (c) ROC curve analysis in plasma biomarkers; (d) Box-and-whisker plots of plasma biomarkers. Statistical significance was assessed using independent *t*-tests. (* *p* < 0.05, ** *p* < 0.01).

In the hippocampus, glutamic acid (AUC = 0.933) and sphingosine (AUC = 0.833) were identified as potential biomarkers, each showing outstanding and good discrimination (Fig. 5a) and exhibiting a significant correlation with the phenotypic data (Supplementary Figure S3a). The box-and-whisker plots further indicated that glutamic acid and sphingosine levels, which were altered by CRS, exhibited a recovery trend following SRI treatment (Fig. 5b). This suggested a significant reversal of CRS-induced metabolic disruptions, as evidenced by the statistical significance of glutamic acid (*p* = 0.019) and sphingosine (*p* = 0.037). In the plasma, methionine (AUC = 0.972), 5-hydroxytryptophan (AUC = 0.833), and valine (AUC = 0.806) were identified as potential biomarkers, showing methionine as an outstanding biomarker, 5-hydroxytryptophan and valine as good discriminators (Fig. 5c), and exhibiting a significant correlation with the phenotypic data (Supplementary Figure S3b). Box-and-whisker plots further revealed that these plasma biomarkers also showed recovery trends following SRI treatment (Fig. 5d), with methionine (*p* = 0.001) and 5-hydroxytryptophan (*p* = 0.045) displaying statistically significant alterations under SRI treatment.

## 4 Discussion

In our previous study, exposure to soil enriched with *S. rimosus* was shown to attenuate depression-like behaviors in a CRS mouse model [29]. Mice were subjected to CRS for 14 days, and direct soil exposure (with or without *S. rimosus*) was provided over a 17-day period spanning the CRS period and immediately before behavior tests. The *S. rimosus*-exposed group exhibited reduced activation of microglia and astrocytes, lower mRNA expression of inflammatory cytokines, and enhanced synaptic plasticity markers compared with both CRS and sterilized-soil groups, which did not show these effects. Importantly, the lack of effect in the sterilized-soil group underscores the role of live microbial components rather than soil exposure, supporting the microbial-origin hypothesis for behavioral and neurobiological benefits.

In a follow-up study, we investigated the effects of exposure to *S. rimosus*-inoculated soil on anxiety-like behaviors and metabolite change in CRS-induced mice. Firstly, behavioral tests, including LDB, EPM, NSFT, and OFT, revealed that CRS induced anxiety-like behaviors, as evidenced by reduced locomotor activity and time spent in the light box and open arms, as well as increased latency to feed. However, exposure to *S. rimosus*-inoculated soil significantly mitigated these behavioral changes, suggesting a potential anxiolytic effect.

To further explore the underlying mechanisms, we examined the neuroinflammatory markers and stress-related neuroendocrine responses in the hippocampus and hypothalamus. CRS increased Iba-1 and GFAP expression in the hippocampus, indicating microglial activation and astrocytic reactivity, respectively. Notably, exposure to *S. rimosus*-inoculated soil reduced the expression of both markers, with a significant reduction in GFAP and a decreasing trend in Iba-1, suggesting an anti-inflammatory effect. Additionally, we analyzed the expression of GR protein and POMC mRNA in the hypothalamus to assess the regulation of the HPA axis. CRS reduces GR expression and increases POMC mRNA levels, indicating a dysregulated stress response. *S. rimosus*-inoculated soil exposure reversed these changes, restoring GR levels and reducing POMC expression, implying a modulatory effect on HPA axis activity.

Common alteration patterns in several metabolite classes were observed across both the hippocampus and plasma. Alterations in amino acids and their derivatives may indicate potential disruptions in neurotransmitter pathways [30]. Changes in lipids and fatty acid derivatives could be associated with mood disorders through various mechanisms, including biological stress responses and inflammation [31]. In addition, changes in nucleosides and their derivatives may result from stress-induced disturbances in purine metabolism and signaling pathways [32]. Studies have implicated carnitines and their derivatives in oxidative stress, suggesting that they may contribute to dysfunction in the central or peripheral nervous system [33]. Overall, our findings suggest that CRS may induce broad metabolic dysregulation at central and peripheral levels. Furthermore, *S. rimosus* exposure may partially restore some of these alterations, which could be associated with the metabolic signatures of stress and potential therapeutic response.

In this study, amino acid metabolism, particularly alanine, aspartate, and glutamate metabolism, and phenylalanine, tyrosine, and tryptophan biosynthesis, exhibited significant alterations in response to anxiety and recovery, which were identified as key pathways in both the hippocampus and plasma. Amino acids are essential for many biological functions, serve as fundamental components of peptides and proteins, and play a crucial role in metabolism [34], [35]. Increasing evidence highlights abnormalities in amino acid levels in the biofluids of patients with stress-related depression compared with healthy controls [36]. Disorders in amino acid metabolism are also prominent in brain tissue, reflecting impaired neural activity [37]. Dysfunction of amino acid metabolism in the brain has been identified in various anxiety models, including CRS models, the prefrontal cortex of learned helpless rats, and chronic unpredictable mild stress models [38], [39], [40]. Furthermore, human studies indicate that alterations in phenylalanine, tryptophan, and tyrosine metabolism are associated with depressive symptoms and changes in functional brain network [41]. In addition, plasma metabolomics in major depressive disorder patients have revealed prominent disruptions in glycine and serine metabolism [42]. Collectively, these findings suggest that the amino acid metabolic disturbances observed in our study reflect key alterations seen in human psychiatric disorders and may serve as potential mechanistic links between anxiety and metabolic dysregulation. Moreover, recovery in these pathways could indicate a therapeutic response.

Glutamate is the primary excitatory neurotransmitter in the central nervous system and plays crucial roles in synaptic transmission, neuroplasticity, and overall brain function. The dysregulation of glutamatergic signaling has been closely linked to the development and progression of depressive disorders, particularly through its involvement in the neurotrophic hypothesis of major depressive disorders (MDD), which emphasizes impaired neuroplasticity and neuronal atrophy [43]. Elevated glutamate levels have been observed in brain regions implicated in emotional regulation, including the hippocampus, in both chronic mild stress and CRS models of anxiety [44], [45]. Excessive glutamate can lead to excitotoxicity by overactivating N-methyl-D-aspartate receptors, particularly in the subgenual cingulate cortex, thereby contributing to synaptic dysfunction and the pathophysiology of anxiety [46]. Consistent with these findings, our study demonstrated increased levels of glutamate in the hippocampi of rats subjected to CRS, supporting its role as a stress-related metabolite. Importantly, glutamate levels significantly decreased following SRI treatment, suggesting the potential recovery of excitatory neurotransmission. Furthermore, glutamate has been identified as a potential biomarker that is responsive to SRI, reinforcing its therapeutic relevance to anxiety-associated metabolic alterations.

Sphingolipids are essential components of biological membranes that play critical roles in signal transduction [47]. Among the sphingolipids, ceramide and sphingomyelin are the most abundant. Ceramides are a group of compounds with similar molecular structures, properties, and functions, comprising complex esters of higher aliphatic alcohols (sphingosine) and fatty acids. Ceramides are broken down into sphingosine, which is subsequently phosphorylated to produce sphingosine 1-phosphate (S1P) [48]. Elevated ceramide levels have been observed in the hippocampus during chronic unpredictable stress, and ceramide accumulation has been implicated in the pathophysiology of MDD [49], [50]. Additionally, chronic immobilization stress in mice has been associated with increased serum S1P levels, whereas chronic unpredictable stress has been linked to significantly elevated S1P levels in brain tissues [51], [52]. These findings highlight the involvement of sphingolipid metabolism in anxiety-related neuropathology, with hippocampal sphingosine emerging as a potential biomarker that reflects alterations in ceramide-S1P signaling under depressive conditions.

Overall, this study demonstrated that short-term exposure to *S. rimosus*–inoculated soil may ameliorate anxiety in a CRS mouse model by modulating amino acid metabolic pathways. It also restored hippocampal and plasma levels of potential anxiety-related biomarkers. Although the present findings provide potential mechanistic insights into amino acid metabolism and stress-related metabolic alterations, they should be interpreted with caution due to species differences and the artificial nature of the restraint stress model. Therefore, the results should be considered within the interpretative limitations of a proof-of-concept animal study. Nevertheless, the relatively small sample size (n = 6 per group) limits statistical power and increases variability, thereby reducing the robustness and generalizability of the conclusions. While the consistency observed across behavioral outcomes and metabolomic alterations lends support to our findings, these results should be interpreted with caution, and larger-scale studies with independent replication will be required to validate and extend these observations.

In addition, the immediate exposure before behavioral testing raises the possibility that volatile organic compounds (VOCs) emitted by *S. rimosus* contributed to the observed effects via olfactory pathways. We regard this not only a potential confounder but also a plausible mechanistic explanation that warrants further investigation. Future studies should therefore implement controlled exposure paradigms and conduct targeted analyses of olfactory receptor activation and associated neural circuits. Such approaches will be essential to disentangle the specific role of olfactory stimulation from systemic mechanisms and to evaluate the translational potential of soil microbiota–derived interventions for stress-related disorders.

## 5 Conclusion

In this study, anxiety-alleviation effects induced by short-term exposure to *S. rimosus* were evaluated using a CRS-induced mouse model in behavioral and tissue analyses and metabolic alterations with an untargeted metabolomic approach on the hippocampus and plasma samples.

Overall, these findings suggest that exposure to *S. rimosus*-inoculated soil alleviates CRS-induced anxiety-like behaviors, potentially through the suppression of neuroinflammation and normalization of the HPA axis function. Metabolomic approaches, including multivariate analysis, heatmap analysis, pathway analysis, ROC curves, and correlation analysis were conducted to identify altered metabolites and metabolic pathways, identify potential biomarkers for SRI treatment, and determine metabolites significantly correlated with phenotypic data. Multivariate analysis revealed distinct metabolic profiles in both hippocampus and plasma between the groups. A total of 83 hippocampal and 92 plasma differential metabolites were identified, reflecting changes in metabolic pathways. Amino acid metabolism pathways linked to impaired neural activity in anxiety were significantly affected by both CRS and SRI treatments. Notably, alanine, aspartate, and glutamate metabolism, as well as phenylalanine, tyrosine, and tryptophan biosynthesis, were altered in both hippocampal and plasma samples. In the hippocampus, glutamate and sphingosine were proposed as biomarkers, showing normalized levels in the SRI group and significant correlations with phenotypic data. In plasma, 5-hydroxytryptophan, methionine, and valine were identified as potential biomarkers, demonstrating normalization in the SRI group and a significant correlation with the phenotype.

In conclusion, this study demonstrated that short-term exposure to *S. rimosus* inoculated soil may ameliorate anxiety in a CRS mouse model by modulating amino acid metabolic pathways and restoring the hippocampal and plasma levels of potential anxiety-related biomarkers. However, because this was a basic study with a relatively small sample size, its generalizability remains limited. Further studies with larger sample sizes are required to validate and strengthen these findings. Furthermore, to clarify the independent role of volatile compounds emitted by *S. rimosus* and their anxiolytic effects via the olfactory system, future studies should implement controlled exposure settings and conduct targeted analyses of olfactory receptor activation and associated neural pathways. It is essential to distinguish the specific contributions of olfactory stimulation to the observed outcomes.

## Author contributions

S. Y. performed soil preparation, bacterial incubation, metabolomic analysis and correlation analysis. J. H. K. performed the animal experiments, behavioral tests, and tissue analysis. S. Y. and J. H. K. wrote the main manuscript. C. H. L and M. S. O. reviewed and edited the manuscript.

## CRediT authorship contribution statement

**Sowon Yang:** Writing – original draft, Visualization, Investigation, Formal analysis. **Jin Hee Kim:** Writing – original draft, Visualization, Investigation, Formal analysis. **Sin-Ae Park:** Resources. **Myung Sook Oh:** Writing – review & editing, Conceptualization. **Choong Hwan Lee:** Writing – review & editing, Conceptualization.

## Code availability

No custom code was used in this study.

## Declaration of Competing Interest

The authors declare no competing interests

## Data Availability

The datasets generated and/or analyzed during the current study are available from the corresponding author on reasonable request.

## References

[1] Levine J.A., McCrady S.K., Boyne S., Smith J., Cargill K., Forrester T. (2011). Non-exercise physical activity in agricultural and urban people. Urban Stud.

[2] Delisle H., Ntandou-Bouzitou G., Agueh V., Sodjinou R., Fayomi B. (2012). Urbanisation, nutrition transition and cardiometabolic risk: the Benin study. Br J Nutr.

[3] Bedrosian T.A., Nelson R.J. (2013). Influence of the modern light environment on mood. Mol Psychiatry.

[4] Cyril S., Oldroyd J.C., Renzaho A. (2013). Urbanisation, urbanicity, and health: a systematic review of the reliability and validity of urbanicity scales. BMC Public Health.

[5] Ng S.W., Howard A., Wang H.J., Su C., Zhang B. (2014). The physical activity transition among adults in China: 1991–2011. Obes Rev.

[6] Zhai F.Y., Du S.F., Wang Z.H., Zhang J.G., Du W.W., Popkin B.M. (2014). Dynamics of the Chinese diet and the role of urbanicity, 1991–2011. Obes Rev.

[7] Logan A.C., Jacka F.N. (2014). Nutritional psychiatry research: an emerging discipline and its intersection with global urbanization, environmental challenges and the evolutionary mismatch. J Physiol Anthr.

[8] Ventriglio A., Torales J., Castaldelli-Maia J.M., De Berardis D., Bhugra D. (2021). Urbanization and emerging mental health issues. CNS Spectr.

[9] Fuller R.A., Irvine K.N., Devine-Wright P., Warren P.H., Gaston K.J. (2007). Psychological benefits of greenspace increase with biodiversity. Biol Lett.

[10] Dallimer M., Irvine K.N., Skinner A.M.J., Davies Z.G., Rouquette J.R., Maltby L.L. (2012). Biodiversity and the feel-good factor: understanding associations between self-reported human well-being and species richness. Bioscience.

[11] Cox D.T.C., Shanahan D.F., Hudson H.L., Fuller R.A., Gaston K.J. (2018). The impact of urbanisation on nature dose and the implications for human health. Land Urban Plan.

[12] Cox D.T.C., Shanahan D.F., Hudson H.L., Plummer K.E., Siriwardena G.M., Fuller R.A. (2017). Doses of neighborhood nature: the benefits for mental health of living with nature. AIBS Bull.

[13] Li Z., Zhang W., Cui J., Liu H., Liu H. (2024). Beneficial effects of short-term exposure to indoor biophilic environments on psychophysiological health: evidence from electrophysiological activity and salivary metabolomics. Environ Res.

[14] Lopes M.K.S., Falk T.H. (2024). Audio-visual-olfactory immersive digital nature exposure for stress and anxiety reduction: a systematic review on systems, outcomes, and challenges. Front Virtual Real.

[15] Kutlu A.K., Yılmaz E., Çeçen D. (2008). Effects of aroma inhalation on examination anxiety. Teach Learn Nurs.

[16] Touhara K., Vosshall L.B. (2009). Sensing odorants and pheromones with chemosensory receptors. Annu Rev Physiol.

[17] Breer H. (2003). Sense of smell: recognition and transduction of olfactory signals. Biochem Soc Trans.

[18] Strous R.D., Shoenfeld Y. (2006). To smell the immune system: olfaction, autoimmunity and brain involvement. Autoimmun Rev.

[19] Insam H., Seewald M.S.A. (2010). Volatile organic compounds (VOCs) in soils. Biol Fertil Soils.

[20] Raza W., Mei X., Wei Z., Ling N., Yuan J., Wang J. (2017). Profiling of soil volatile organic compounds after long-term application of inorganic, organic and organic-inorganic mixed fertilizers and their effect on plant growth. Sci Total Environ.

[21] Schöller C.E.G., Gürtler H., Pedersen R., Molin S., Wilkins K. (2002). Volatile metabolites from actinomycetes. J Agric Food Chem.

[22] Rabe P., Citron C.A., Dickschat J.S. (2013). Volatile terpenes from actinomycetes: a biosynthetic study correlating chemical analyses to genome data. ChemBioChem.

[23] Lin T.-F., Wong J.-Y., Kao H.-P. (2002). Correlation of musty odor and 2-MIB in two drinking water treatment plants in south Taiwan. Sci Total Environ.

[24] Liato V., Aïder M. (2017). Geosmin as a source of the earthy-musty smell in fruits, vegetables and water: origins, impact on foods and water, and review of the removing techniques. Chemosphere.

[25] Kim S.O., Kim M.J., Choi N.Y., Kim J.H., Oh M.S., Lee C.H. (2022). Psychophysiological and metabolomics responses of adults during horticultural activities using soil inoculated with streptomyces rimosus: a pilot study. Int J Environ Res Public Health.

[26] Kim R., Yang S., Lee C.H., Park S.-A. (2025). Horticultural activity in soil inoculated with streptomyces rimosus improved depressive mood with altered electroencephalogram and serum metabolism in adults. Sci Rep.

[27] Zhao X., Seese R.R., Yun K., Peng T., Wang Z. (2013). The role of galanin system in modulating depression, anxiety, and addiction-like behaviors after chronic restraint stress. Neuroscience.

[28] Jun B.-G., Kim S.-H., Kim S.-H., Hong S.-M., Lee H., Lim Y. (2024). Metabolomic comparison of guava (Psidium guajava L.) leaf extracts fermented by limosilactobacillus fermentum and lactiplantibacillus plantarum and their antioxidant and antiglycation activities. Nutrients.

[29] Kim J.H., Kim J.S., Eo H., Yang S., Lee C.H., Park S.-A. (2025). Streptomyces rimosus-rich soil exposure alleviates depression-like behaviors by modulating neuroinflammation and synaptic plasticity in mice with stress. Sci Rep.

[30] Teleanu R.I., Niculescu A.-G., Roza E., Vladâcenco O., Grumezescu A.M., Teleanu D.M. (2022). Neurotransmitters—key factors in neurological and neurodegenerative disorders of the central nervous system. Int J Mol Sci.

[31] Mocking R.J.T., Assies J., Ruhé H.G., Schene A.H. (2018). Focus on fatty acids in the neurometabolic pathophysiology of psychiatric disorders. J Inherit Metab Dis.

[32] Tomczyk M., Glaser T., Slominska E.M., Ulrich H., Smolenski R.T. (2021). Purine nucleotides metabolism and signaling in Huntington’s disease: search for a target for novel therapies. Int J Mol Sci.

[33] Ferreira G.C., McKenna M.C. (2017). L-carnitine and acetyl-L-carnitine roles and neuroprotection in developing brain. Neurochem Res.

[34] Guevara-Cruz M., Vargas-Morales J.M., Méndez-García A.L., López-Barradas A.M., Granados-Portillo O., Ordaz-Nava G. (2018). Amino acid profiles of young adults differ by sex, body mass index and insulin resistance. Nutr Metab Cardiovasc Dis.

[35] Dai Z., Zheng W., Locasale J.W. (2022). Amino acid variability, tradeoffs and optimality in human diet. Nat Commun.

[36] Hung C.-I., Lin G., Chiang M.-H., Chiu C.-Y. (2021). Metabolomics-based discrimination of patients with remitted depression from healthy controls using ^1^H NMR spectroscopy. Sci Rep.

[37] Ni Y., Su M., Lin J., Wang X., Qiu Y., Zhao A. (2008). Metabolic profiling reveals disorder of amino acid metabolism in four brain regions from a rat model of chronic unpredictable mild stress. FEBS Lett.

[38] Chen G., Yang D., Yang Y., Li J., Cheng K., Tang G. (2015). Amino acid metabolic dysfunction revealed in the prefrontal cortex of a rat model of depression. Behav Brain Res.

[39] Zhou X., Liu L., Zhang Y., Pu J., Yang L., Zhou C. (2017). Metabolomics identifies perturbations in amino acid metabolism in the prefrontal cortex of the learned helplessness rat model of depression. Neuroscience.

[40] Tian L., Pu J., Liu Y., Gui S., Zhong X., Song X. (2020). Metabolomic analysis of animal models of depression. Metab Brain Dis.

[41] Zhao S., Khoo S., Ng S.-C., Chi A. (2022). Brain functional network and amino acid metabolism association in females with subclinical depression. Int J Environ Res Public Health.

[42] Du Y., Wei J., Zhang Z., Yang X., Wang M., Wang Y. (2021). Plasma metabolomics profiling of metabolic pathways affected by major depressive disorder. Front Psychiatry.

[43] Duan J., Xie P. (2020). The potential for metabolomics in the study and treatment of major depressive disorder and related conditions. Expert Rev Proteom.

[44] Liu L., Zhou X., Zhang Y., Liu Y., Yang L., Pu J. (2016). The identification of metabolic disturbances in the prefrontal cortex of the chronic restraint stress rat model of depression. Behav Brain Res.

[45] Akimoto H., Oshima S., Sugiyama T., Negishi A., Nemoto T., Kobayashi D. (2019). Changes in brain metabolites related to stress resilience: metabolomic analysis of the hippocampus in a rat model of depression. Behav Brain Res.

[46] McCarthy D.J., Alexander R., Smith M.A., Pathak S., Kanes S., Lee C.-M. (2012). Glutamate-based depression GBD. Med Hypotheses.

[47] Babenko N.A., Shevereva V.M., Gar’kavenko V.V. (2016). Effects of chronic neurogenic stress on behavior of rats and contents of sphingolipids in their brain and peripheral tissues. Neurophysiology.

[48] van Kruining D., Luo Q., van Echten-Deckert G., Mielke M.M., Bowman A., Ellis S. (2020). Sphingolipids as prognostic biomarkers of neurodegeneration, neuroinflammation, and psychiatric diseases and their emerging role in lipidomic investigation methods. Adv Drug Deliv Rev.

[49] Jernigan P.L., Hoehn R.S., Grassmé H., Edwards M.J., Müller C.P., Kornhuber J. (2015). Sphingolipids in major depression. Neurosignals.

[50] Oliveira T.G., Chan R.B., Bravo F.V., Miranda A., Silva R.R., Zhou B. (2016). The impact of chronic stress on the rat brain lipidome. Mol Psychiatry.

[51] Jang S., Kim D., Lee Y., Moon S., Oh S. (2011). Modulation of sphingosine 1-phosphate and tyrosine hydroxylase in the stress-induced anxiety. Neurochem Res.

[52] Fischer C., Thomas D., Gurke R., Tegeder I. (2024). Brain region specific regulation of anandamide (down) and sphingosine-1-phosphate (up) in association with anxiety (AEA) and resilience (S1P) in a mouse model of chronic unpredictable mild stress. Pflügers ArchEur J Physiol.

