# Supplementary material for: Streptomyces rimosus-inoculated soil exposure modulates metabolomic profiles under chronic stress
Source: Comput Struct Biotechnol J. 2025 Oct 17;27:4431–40. doi: 10.1016/j.csbj.2025.10.030 (PMC12589958; doi:10.1016/j.csbj.2025.10.030)
Supplement: Supplementary file 1 — Supplementary material [file mmc1.docx]

**Supplementary Table 1.** Differential metabolites in hippocampus identified by GC-TOF-MS.

| **No.** | **Tentative Identification^a^** | **RT^b^ (min)** | **VIP 1^c^** | **VIP 2^d^** | **Unique mass (*m/z*)** | **MS fragments (*m/z*)** | **ID** | **TMS^g^** |
| --- | --- | --- | --- | --- | --- | --- | --- | --- |
|  | *Carbohydrates and derivatives* |  |  |  |  |  |  |  |
| 1 | Erythritol | 9.72 | 2.20 | 1.85 | 116 | 117, 116, 103, 217, 69, 101, 205, 204, 189 | MS^e^ | 4 |
| 2 | Sorbitol | 12.97 | 2.36 | 1.93 | 205 | 205, 217, 319, 103, 117, 74, 244, 75 | MS | 6 |
| 3 | Mannose | 15.27 | 2.69 | 1.98 | 387 | 387, 160, 357, 388, 247, 131, 157, 389, 471 | STD^f^ | 6 |
| 4 | Sucrose | 17.01 | 0.95 | 1.36 | 361 | 361, 362, 169, 271, 363, 437, 243, 451 | MS | 8 |
|  | *Amino acids and derivatives* |  |  |  |  |  |  |  |
| 5 | Alanine | 5.50 | 2.16 | 1.66 | 116 | 154, 110, 69, 56, 221, 73, 103, 211, 155, 57 | STD | 0 |
| 6 | Serine | 8.42 | 1.68 | 1.20 | 204 | 204, 218, 205, 219, 206, 278, 75, 220, 306 | STD | 3 |
| 7 | Aspartic acid | 9.80 | 1.62 | 1.16 | 232 | 232, 100, 218, 233, 74, 75, 202 | STD | 3 |
| 8 | Proline | 9.88 | 1.45 | 1.25 | 156 | 156, 157, 258, 230, 74, 158, 75, 122 | MS | 2 |
| 9 | Acetylglutamate | 11.81 | 2.48 | 1.99 | 84 | 84, 158, 156, 216, 56, 126, 186, 174, 318 | STD | 2 |
| 10 | Tyrosine | 12.90 | 1.91 | 1.41 | 218 | 218, 219, 100, 280, 220, 179, 281, 354, 180 | STD | 3 |
|  | *Carboxylic acids and derivatives* |  |  |  |  |  |  |  |
| 11 | Glycolic acid | 5.59 | 1.45 | 1.04 | 73 | 66, 148, 205, 177, 133, 149, 74, 161 | MS | 2 |
| 12 | Malic acid | 9.53 | 2.02 | 1.47 | 73 | 233, 55, 74, 133, 245, 148, 189, 190 | MS | 3 |
| 13 | Citric acid | 12.10 | 1.80 | 1.47 | 273 | 273, 274, 347, 363, 375, 75, 211, 149 | MS | 4 |
|  | *Fatty acids and derivatives* |  |  |  |  |  |  |  |
| 14 | Hexanoic acid | 5.54 | 1.38 | 0.99 | 75 | 75, 173, 117, 131, 132, 76, 174, 74, 61 | MS | 1 |
| 15 | 2-Hydroxy-3-methylvaleric acid | 8.94 | 1.65 | 1.19 | 103 | 103, 75, 159, 117, 116, 69, 74, 131 | MS | 2 |
| 16 | Decanoic acid | 9.21 | 0.60 | 1.60 | 229 | 75, 117, 229, 132, 129, 218, 131, 145, 77 | MS | 1 |
| 17 | Glutaric acid | 10.22 | 2.14 | 1.60 | 247 | 129, 247, 75, 157, 203, 85, 133, 349, 130 | MS | 3 |
| 18 | Lauric acid | 10.77 | 1.47 | 1.51 | 117 | 117, 75, 257, 132, 129, 55, 145, 131 | MS | 1 |
| 19 | Myristic acid | 12.19 | 1.41 | 1.70 | 285 | 117, 75, 132, 285, 129, 55, 145, 79, 74 | MS | 1 |
| 20 | 9-Hexadecenoic acid | 13.38 | 1.21 | 1.36 | 117 | 117, 75, 55, 129, 79, 311, 145, 96, 69, 67 | MS | 1 |
| 21 | Cervonic acid | 16.44 | 1.81 | 1.38 | 73 | 79, 91, 67, 75, 93, 117, 119, 105, 108 | MS | 1 |
| 22 | Monopalmitin | 16.54 | 1.95 | 1.46 | 371 | 371, 57, 55, 129, 71, 239, 203, 372 | MS | 2 |
| 23 | 2-Monostearin | 17.32 | 1.69 | 1.22 | 129 | 129, 218, 103, 75, 131, 219, 55, 130 | MS | 2 |
| 24 | Glyceryl Monostearate | 17.51 | 1.55 | 1.22 | 73 | 399, 57, 129, 55, 71, 103, 117, 203 | MS | 2 |
|  | *Nucleosides and derivatives* |  |  |  |  |  |  |  |
| 25 | Uracil | 8.24 | 1.81 | 1.30 | 245 | 245, 241, 99, 256, 255, 75, 113, 246 | MS | 2 |
| 26 | Thymine | 8.81 | 1.25 | 1.06 | 255 | 255, 113, 270, 204, 256, 117, 257, 120 | MS | 2 |
| 27 | Hypoxanthine | 12.03 | 2.19 | 1.57 | 265 | 265, 280, 266, 267, 206, 281, 84, 125, 193 | MS | 2 |
| 28 | Xanthine | 13.46 | 1.44 | 1.33 | 353 | 353, 368, 354, 294, 355, 279, 369, 100 | MS | 3 |
| 29 | Uridine | 15.99 | 2.28 | 1.67 | 73 | 217, 103, 218, 259, 169, 243, 74, 75 | MS | 3 |
| 30 | Inosine | 16.60 | 2.92 | 2.23 | 73 | 217, 230, 245, 103, 259, 281, 237, 193 | MS | 4 |
|  | *Etcs.* |  |  |  |  |  |  |  |
| 31 | 1,4-Butanediol | 6.48 | 1.96 | 1.60 | 116 | 116, 148, 101, 177, 103, 149, 55, 75 | MS | 2 |
| 32 | Acetamide | 6.82 | 0.10 | 1.41 | 147 | 188, 216, 148, 172, 114, 75, 66, 231 | MS | 2 |
| 33 | Trimethylolpropane | 8.74 | 1.88 | 1.38 | 191 | 191, 157, 103, 75, 192, 129, 170, 74 | MS | 3 |
| 34 | Triethylene glycol | 9.63 | 1.58 | 1.33 | 73 | 117, 116, 103, 74, 101, 161, 59, 66 | MS | 2 |
| 35 | Pantothenic acid | 13.29 | 2.81 | 2.21 | 103 | 103, 117, 291, 157, 75, 247, 201, 55, 159 | MS | 3 |
| 36 | Sulfamic acid | 13.51 | 1.43 | 1.15 | 73 | 298, 290, 300, 299, 210, 191, 100, 74 | MS | 3 |
|  | *N.I.D.^h^* |  |  |  |  |  |  |  |
|  | N.I.D. 1 | 9.10 | 1.01 | 0.73 | 201 | 201, 205, 59, 133, 103, 148, 117, 129 |  |  |
|  | N.I.D. 2 | 11.28 | 2.26 | 1.93 | 174 | 174, 175, 86, 176, 100, 59, 248, 130 |  |  |
|  | N.I.D. 3 | 12.16 | 1.20 | 1.45 | 258 | 258, 160, 259, 156, 130, 144, 59, 346, 260 |  |  |
|  | N.I.D. 4 | 12.40 | 2.29 | 1.67 | 373 | 373, 245, 374, 246, 375, 319, 226, 392, 335 |  |  |
|  | N.I.D. 5 | 12.44 | 0.08 | 1.93 | 75 | 75, 131, 116, 302, 129, 55, 212, 144, 132 |  |  |
|  | N.I.D. 6 | 13.38 | 1.77 | 1.49 | 286 | 286, 287, 160, 130, 117, 59, 116, 230 |  |  |

^a^Identified compounds based on the VIP 1 or 2 value (> 1.0) and *p* (< 0.05) from the PLS-DA model in Figure 3a; ^b^RT, retention time; ^c^VIP value based on PLS1; ^d^VIP value based on PLS2; ^e^Mass spectrum compared with the National Institute of Standards and Technology (NIST) database; ^f^Mass spectrum consistent with that of the standard compounds; ^g^TMS, Trimethylsilyl; ^h^N.I.D., Non-identified

**Supplementary Table 2.** Differential metabolites in hippocampus identified by UHPLC-Orbitrap-MS/MS.

| **No.** | **Tentative identification^a^** | **RT^b^ (min)** | **VIP 1^c^** | **VIP 2^d^** | **Precursor ion (m/z)** | **MS fragments^e^ (m/z)** | **Adduct** | **Exact mass** | **Formula** | **Error (ppm)** |
| --- | --- | --- | --- | --- | --- | --- | --- | --- | --- | --- |
|  | *Amino acids and derivatives* |  |  |  |  |  |  |  |  |  |
| 1 | Histidine | 0.68 | 1.19 | 0.86 | 156.076 | (+) 110.071, 113.963, 95.06 | [M+H]+ | 155.069 | C6H9N3O2 | -0.40 |
| 2 | Lysine | 0.68 | 1.07 | 0.80 | 147.112 | (+) 130.086, 84.08, 131.089 | [M+H]+ | 146.105 | C6H14N2O2 | -0.41 |
| 3 | Laminine | 0.69 | 1.16 | 1.03 | 189.159 | (+) 130.086, 84.081, 60.081 | [M+H]+ | 188.152 | C9H20N2O2 | -0.51 |
| 4 | GABA* | 0.74 | 1.48 | 1.05 | 104.070 | (+) 87.044, 86.06, 105.11 | [M+H]+ | 103.063 | C4H9NO2 | -0.21 |
| 5 | Cystine* | 0.74 | 1.35 | 1.23 | 241.030 | (+) 151.983, 120.011, 122.027 | [M+H]+ | 240.023 | C6H12N2O4S2 | -0.75 |
| 6 | Glutamine* | 0.75 | 1.55 | 1.10 | 147.076 | (+) 130.049, 84.044, 101.071 | [M+H]+ | 146.069 | C5H10N2O3 | -0.51 |
| 7 | Carnitine* | 0.76 | 1.67 | 1.20 | 162.112 | (+) 103.039, 60.081, 163.115 | [M+H]+ | 161.105 | C7H15NO3 | -0.53 |
| 8 | Taurine | 0.77 | 1.20 | 1.04 | 126.022 | (+) 108.011, 44.049, 127.025 | [M+H]+ | 125.014 | C2H7NO3S | -0.41 |
| 9 | Glutamic acid* | 0.77 | 1.22 | 1.39 | 148.060 | (+) 130.05, 84.044, 102.055 | [M+H]+ | 147.053 | C5H9NO4 | -0.50 |
| 10 | Creatine | 0.78 | 1.11 | 0.80 | 132.076 | (+) 90.055, 44.049, 133.08 | [M+H]+ | 131.069 | C4H9N3O2 | -0.51 |
| 11 | N-Acetyl-L-aspartic acid* | 0.87 | 1.48 | 1.04 | 176.055 | (+) 134.044, 88.039, 116.034 | [M+H]+ | 175.048 | C6H9NO5 | -0.57 |
| 12 | Pyroglutamic acid* | 1.05 | 1.40 | 1.04 | 128.035 | (-) 128.035, 129.038, 85.030 | [M-H]- | 129.042 | C5H7NO3 | -2.41 |
| 13 | Phenylalanine | 1.06 | 1.20 | 0.86 | 166.086 | (+) 120.08, 131.049, 149.059 | [M+H]+ | 165.079 | C9H11NO2 | -0.44 |
| 14 | Pantothenic acid* | 1.06 | 1.40 | 0.99 | 220.117 | (+) 90.055, 202.107, 184.096 | [M+H]+ | 219.110 | C9H17NO5 | -0.59 |
| 15 | Tryptophan | 1.48 | 1.23 | 0.87 | 205.096 | (+) 188.07, 146.06, 169.074 | [M+H]+ | 204.089 | C11H12N2O2 | -0.72 |
| 16 | N-Acetyl-L-leucine | 4.02 | 0.55 | 1.17 | 174.112 | (+) 156.102, 139.075, 128.107 | [M+H]+ | 173.105 | C8H15NO3 | -3.14 |
| 17 | 7-Keto-8-aminopelargonic acid | 5.91 | 0.60 | 1.01 | 188.127 | (+) 170.117, 125.096, 142.122 | [M+H]+ | 187.120 | C9H17NO3 | -3.85 |
|  | *Nucleosides and derivatives* |  |  |  |  |  |  |  |  |  |
| 18 | Dihydrothymine | 0.77 | 1.31 | 0.92 | 129.065 | (+) 84.0440, 130.0495, 129.066 | [M+H]+ | 128.058 | C5H8N2O2 | -3.41 |
| 19 | Adenosine monophosphate* | 0.80 | 1.45 | 1.08 | 348.069 | (+) 136.061, 268.89, 250.88 | [M+H]+ | 347.062 | C10H14N5O7P | -1.15 |
| 20 | Adenosylhomocysteine | 0.84 | 0.94 | 1.07 | 385.128 | (+) 134.027, 136.061, 250.074 | [M+H]+ | 384.121 | C14H20N6O5S | -0.97 |
| 21 | Hypoxanthine* | 0.86 | 1.19 | 1.14 | 137.045 | (+) 138.049, 110.035, 119.035 | [M+H]+ | 136.038 | C5H4N4O | -0.45 |
|  | *Lipids and derivatives* |  |  |  |  |  |  |  |  |  |
| 22 | Phosphocholine* | 0.76 | 1.59 | 1.25 | 184.073 | (+) 86.096, 124.999, 60.081 | [M+H]+ | 183.065 | C5H14NO4P | -0.69 |
| 23 | Acetyl carnitine | 1.31 | 0.75 | 1.15 | 204.123 | (+) 85.028, 145.049, 60.081 | [M+H]+ | 203.115 | C9H17NO4 | -0.44 |
| 24 | Lauryl diethanolamide | 8.91 | 1.04 | 0.74 | 288.252 | (+) 242.247, 264.872, 236.877 | [M+H]+ | 287.245 | C16H33NO3 | -0.85 |
| 25 | N-(2-Hydroxyethyl)tetradecanamide | 9.17 | 1.06 | 0.75 | 272.258 | (+) 254.247, 255.251, 100.075 | [M+H]+ | 271.250 | C16H33NO2 | 0.61 |
| 26 | LysoPE(16:0) | 10.73 | 1.04 | 0.75 | 452.277 | (-) 255.232, 256.236, 196.038 | [M-H]- | 453.285 | C21H44NO7P | -2.13 |
| 27 | LysoPC(16:0) | 10.79 | 1.09 | 0.81 | 496.338 | (+) 184.073, 104.107, 478.328 | [M+H]+ | 495.331 | C24H50NO7P | -1.39 |
| 28 | LPE 18:1 | 11.01 | 1.17 | 0.83 | 478.293 | (-) 281.248, 196.038, 214.048 | [M-H]- | 479.300 | C23H46NO7P | -1.49 |
| 29 | Sphingosine | 11.09 | 0.29 | 1.39 | 300.289 | (+) 282.279, 299.349, 247.242 | [M+H]+ | 299.282 | C18H37NO2 | -0.80 |
| 30 | LPE O-18:2* | 11.27 | 1.36 | 1.00 | 462.298 | (-) 196.038, 140.012, 265.253 | [M-H]- | 463.305 | C23H46NO6P | -1.73 |
| 31 | LPE O-18:1 | 12.20 | 1.16 | 0.82 | 464.314 | (-) 196.038, 140.012, 267.269 | [M-H]- | 465.321 | C23H48NO6P | -2.47 |
| 32 | Oleoylethanolamine | 12.49 | 0.07 | 1.23 | 326.304 | (+) 62.06, 326.377, 97.101 | [M+H]+ | 325.297 | C20H39NO2 | -1.00 |
| 33 | Erucamide | 14.03 | 1.15 | 0.92 | 338.341 | (+) 83.085, 97.101, 57.07 | [M+H]+ | 337.333 | C22H43NO | -1.07 |
| 34 | Lipid 1 | 9.77 | 0.60 | 1.28 | 344.242 | (+) 172.133, 158.117, 218.138 | [M+H]+ | 343.235 |  |  |
| 35 | Lipid 2 | 9.81 | 1.06 | 0.75 | 387.192 | (+) 331.130, 175.015, 276.067 | [M+H]+ | 386.185 |  |  |
| 36 | Lipid 3 | 10.36 | 1.02 | 0.72 | 548.273 | (+) 505.231, 407.255, 385.273 | [M+H]+ | 547.266 |  |  |
| 37 | Lipid 4 | 12.98 | 1.20 | 0.89 | 637.303 | (+) 581.241, 525.179, 393.085 | [M+H]+ | 636.296 |  |  |
| 38 | Lipid 5* | 13.23 | 0.57 | 1.55 | 398.362 | (+) 142.122, 160.1323, 399.307 | [M+H]+ | 397.355 |  |  |
|  | *Etcs.* |  |  |  |  |  |  |  |  |  |
| 39 | Carnosine* | 0.67 | 1.32 | 0.98 | 227.113 | (+) 156.076, 110.071, 210.087 | [M+H]+ | 226.106 | C9H14N4O3 | -0.66 |
| 40 | Homocarnosine | 0.67 | 1.25 | 1.02 | 241.129 | (+) 156.076, 110.071, 86.060 | [M+H]+ | 240.122 | C10H16N4O3 | -2.91 |
| 41 | N-Acetylneuraminic acid | 0.80 | 1.26 | 0.96 | 310.112 | (+) 167.033, 274.091, 197.044 | [M+H]+ | 309.105 | C11H19NO9 | -1.08 |
| 42 | D-Glucose 6-phosphate* | 0.80 | 1.49 | 1.56 | 259.022 | (-) 96.970, 78.959, 138.980 | [M-H]- | 260.029 | C6H13O9P | -1.71 |
| 43 | Indoline | 1.06 | 1.14 | 0.82 | 120.080 | (+) 121.083, 103.053, 93.069 | [M+H]+ | 119.073 | C8H9N | -2.32 |
| 44 | Spaglumic acid* | 1.06 | 1.40 | 1.03 | 305.097 | (+) 148.06, 130.05, 253.88 | [M+H]+ | 304.090 | C11H16N2O8 | -0.90 |
| 45 | Pentaethylene glycol | 1.09 | 1.05 | 0.76 | 239.148 | (+) 89.059, 133.086, 203.046 | [M+H]+ | 238.141 | C10H22O6 | -0.69 |
| 46 | Tributoxyethyl phosphate | 11.43 | 1.05 | 0.79 | 399.249 | (+) 199.072, 57.07, 83.085 | [M+H]+ | 398.242 | C18H39O7P | -1.42 |
| 47 | Dioctyl Phthalate | 13.89 | 1.15 | 0.82 | 391.283 | (+) 149.023, 71.085, 89.06 | [M+H]+ | 390.276 | C24H38O4 | -1.27 |
|  | *N.I.D.^f^* |  |  |  |  |  |  |  |  |  |
|  | N.I.D. 1 | 0.71 | 1.22 | 0.86 | 226.951 | (+) 90.976, 158.964, 156.076 |  |  |  |  |
|  | N.I.D. 2* | 0.71 | 1.37 | 0.97 | 378.899 | (+) 106.950, 174.938, 90.976 |  |  |  |  |
|  | N.I.D. 3 | 0.71 | 1.05 | 0.82 | 190.911 | (+) 122.924, 167.977, 191.040 |  |  |  |  |
|  | N.I.D. 4 | 0.77 | 0.69 | 1.04 | 251.036 | (+) 126.022, 119.089, 118.086 |  |  |  |  |
|  | N.I.D. 5* | 1.19 | 1.35 | 0.96 | 223.117 | (+) 99.044, 73.028, 117.054 |  |  |  |  |
|  | N.I.D. 6 | 1.19 | 1.23 | 0.87 | 245.099 | (+) 246.102, 222.931, 200.036 |  |  |  |  |
|  | N.I.D. 7* | 1.51 | 1.39 | 0.98 | 154.041 | (+) 118.065, 119.073, 113.963 |  |  |  |  |
|  | N.I.D. 8* | 1.51 | 1.42 | 1.00 | 202.043 | (+) 156.038, 155.045, 157.042 |  |  |  |  |
|  | N.I.D. 9* | 1.52 | 1.95 | 1.49 | 299.971 | (+) 154.041, 120.081, 209.976 |  |  |  |  |
|  | N.I.D. 10 | 5.91 | 0.55 | 1.02 | 142.122 | (+) 113.963, 97.101, 125.096 |  |  |  |  |
|  | N.I.D. 11 | 5.91 | 0.48 | 1.06 | 210.109 | (+) 211.113, 200.918, 81.033 |  |  |  |  |
|  | N.I.D. 12* | 5.91 | 1.81 | 1.28 | 283.073 | (+) 260.057, 242.047, 282.278 |  |  |  |  |
|  | N.I.D. 13 | 7.16 | 0.64 | 1.11 | 198.148 | (+) 135.117, 198.148, 152.143 |  |  |  |  |
|  | N.I.D. 14 | 7.16 | 0.54 | 1.04 | 216.159 | (+) 198.148, 135.117, 153.127 |  |  |  |  |
|  | N.I.D. 15* | 9.16 | 0.68 | 1.48 | 312.252 | (+) 277.216, 295.226, 123.117 |  |  |  |  |
|  | N.I.D. 16 | 9.71 | 0.24 | 1.01 | 338.266 | (+) 339.269, 337.254, 97.101 |  |  |  |  |
|  | N.I.D. 17 | 11.02 | 0.49 | 1.13 | 361.269 | (+) 81.070, 95.085, 245.226 |  |  |  |  |
|  | N.I.D. 18 | 11.23 | 1.14 | 0.83 | 249.184 | (+) 193.122, 250.188, 137.059 |  |  |  |  |
|  | N.I.D. 19 | 11.59 | 0.04 | 1.57 | 467.383 | (+) 170.117, 89.060, 133.086 |  |  |  |  |

^a^Identified compounds based on the VIP 1 or 2 value (> 1.0) from the PLS-DA model in Figure 3b; ^b^RT, retention time; ^c^VIP value based on PLS1; ^d^VIP value based on PLS2; ^e^Mass fragment patterns detected in positive mode; ^f^N.I.D, Non-identified (* *p* < 0.05)

**Supplementary Table 3.** Differential metabolites in plasma identified by GC-TOF-MS.

| **No.** | **Tentative Identification^a^** | **RT^b^ (min)** | **VIP 1^c^** | **VIP 2^d^** | **Unique mass (*m/z*)** | **MS fragments (*m/z*)** | **ID** | **TMS^g^** |
| --- | --- | --- | --- | --- | --- | --- | --- | --- |
|  | *Carbohydrates and derivatives* |  |  |  |  |  |  |  |
| 1 | Arabinose | 10.98 | 3.23 | 2.42 | 103 | 103, 217, 307, 75, 74, 104, 189 | STD^e^ | 4 |
| 2 | Xylose | 11.09 | 1.45 | 1.95 | 103 | 103, 205, 217, 117, 74, 75, 307, 173 | STD | 4 |
| 3 | Xylitol | 11.32 | 1.00 | 1.83 | 103 | 103, 217, 205, 129, 307, 319, 218, 191 | STD | 5 |
| 4 | Rhamnose | 11.49 | 2.20 | 2.89 | 117 | 117, 160, 118, 75, 129, 74, 219, 277 | STD | 4 |
| 5 | Galactose | 12.62 | 0.00 | 2.48 | 319 | 205, 319, 160, 103, 217, 320, 117, 74 | STD | 5 |
| 6 | Sorbitol | 12.97 | 0.94 | 1.98 | 319 | 319, 205, 103, 217, 320, 117, 307, 206 | STD | 6 |
| 7 | Glucuronic acid | 16.47 | 1.47 | 1.22 | 217 | 217, 204, 218, 233, 74, 131, 143, 205 | MS^f^ | 5 |
| 8 | Lactose | 17.58 | 1.63 | 1.29 | 361 | 361, 217, 103, 362, 191, 169, 363, 271 | STD | 8 |
|  | *Amino acids and derivatives* |  |  |  |  |  |  |  |
| 9 | Glycine | 7.92 | 2.84 | 2.13 | 174 | 174, 86, 175, 248, 100, 59, 176, 74 | STD | 3 |
| 10 | Aspartic acid | 9.43 | 2.53 | 2.22 | 232 | 70, 232, 185, 100, 233, 200, 157, 77 | STD | 3 |
| 11 | Pyroglutamic acid | 9.84 | 0.93 | 1.45 | 156 | 156, 157, 230, 258, 75, 158, 122, 148 | STD | 2 |
| 12 | Phenylalanine | 10.10 | 1.70 | 2.00 | 120 | 120, 146, 75, 91, 130, 103, 74, 121 | STD | 1 |
| 13 | Proline | 10.32 | 2.53 | 1.92 | 142 | 142, 186, 143, 216, 75, 74, 187, 288 | STD | 2 |
| 14 | Asparagine | 10.88 | 1.86 | 1.52 | 73 | 75, 158, 74, 117, 160, 129, 116 | STD | 3 |
| 15 | Tyrosine | 12.56 | 2.93 | 2.78 | 179 | 179, 180, 75, 208, 146, 74, 181, 91 | STD | 2 |
| 16 | Tryptophan | 14.46 | 1.85 | 1.36 | 202 | 202, 203, 127, 233, 52, 128, 158, 377 | STD | 3 |
| 17 | 5-Hydroxytryptophan | 16.07 | 1.73 | 1.30 | 290 | 202, 290, 191, 203, 100, 130, 291, 74 | STD | 3 |
|  | *Carboxylic acids and derivatives* |  |  |  |  |  |  |  |
| 18 | 2-Methoxymandelic acid | 5.30 | 2.67 | 1.97 | 209 | 75, 209, 74, 179, 59, 148, 119, 146 | MS | 2 |
| 19 | Lactic acid | 5.34 | 1.62 | 1.66 | 117 | 117, 66, 191, 74, 190, 75, 88 | MS | 2 |
| 20 | Oxalic acid | 6.12 | 1.00 | 2.05 | 73 | 148, 66, 72, 74, 190, 59, 149, 219 | MS | 2 |
| 21 | Fumaric acid | 8.23 | 2.79 | 2.33 | 73 | 245, 75, 99, 241, 246, 143, 148, 256 | MS | 2 |
| 22 | Benzylacetic acid | 8.94 | 1.69 | 2.04 | 104 | 104, 75, 207, 91, 52, 79, 105, 222 | MS | 1 |
| 23 | Malic acid | 9.52 | 1.03 | 1.67 | 245 | 233, 75, 55, 74, 133, 245, 148, 101 | MS | 3 |
|  | *Fatty acids and derivatives* |  |  |  |  |  |  |  |
| 24 | Hexanoic acid | 5.44 | 2.03 | 1.67 | 173 | 75, 173, 117, 131, 132, 165, 76, 61 | MS | 1 |
| 25 | 2-Hydroxybutyric acid | 6.42 | 0.33 | 2.16 | 233 | 117, 191, 75, 148, 88, 233, 66, 149 | MS | 2 |
| 26 | 2-Hydroxyisovaleric acid | 6.49 | 0.45 | 2.37 | 145 | 145, 146, 148, 219, 133, 149, 59, 55 | STD | 2 |
| 27 | Valeric acid | 6.65 | 0.81 | 2.57 | 73 | 89, 57, 59, 56, 58, 200, 75, 189 | MS | 1 |
| 28 | 2-Hydroxyisocaproic acid | 7.20 | 2.92 | 2.50 | 159 | 103, 159, 69, 75, 160, 104, 177, 233 | STD | 2 |
| 29 | Octanoic acid | 7.44 | 1.89 | 1.47 | 201 | 75, 117, 201, 132, 55, 129, 131, 74 | MS | 1 |
| 30 | Nonanoic acid | 8.35 | 1.59 | 1.73 | 73 | 75, 117, 215, 132, 129, 55, 131, 74 | MS | 1 |
| 31 | 2-Hydroxyglutaric acid | 10.21 | 0.70 | 2.05 | 247 | 129, 247, 85, 157, 203, 149, 349, 116 | MS | 3 |
| 32 | Glutaric acid | 10.23 | 1.27 | 1.83 | 73 | 75, 55, 74, 198, 156, 89, 59, 186 | MS | 2 |
| 33 | Suberic acid | 11.13 | 1.97 | 2.03 | 83 | 75, 55, 83, 187, 169, 117, 129, 139 | MS | 2 |
| 34 | Myristic acid | 12.18 | 1.56 | 1.45 | 73 | 75, 117, 132, 285, 129, 55, 145, 131 | MS | 1 |
| 35 | 9-Hexadecenoic acid | 13.36 | 0.31 | 1.71 | 75 | 117, 84, 67, 145, 96, 311, 81, 132 | MS | 1 |
| 36 | Heptadecanoic acid | 14.08 | 0.25 | 1.83 | 174 | 75, 117, 132, 174, 129, 145, 55, 327 | MS | 1 |
| 37 | Oleic acid | 14.51 | 0.07 | 1.55 | 67 | 75, 117, 129, 67, 339, 145, 81, 55 | MS | 1 |
| 38 | Stearic acid | 14.66 | 0.61 | 1.87 | 117 | 117, 75, 132, 129, 145, 341, 55, 57 | MS | 1 |
|  | *Lipids and derivatives* |  |  |  |  |  |  |  |
| 39 | Monopalmitin | 16.54 | 0.98 | 1.47 | 371 | 371, 57, 55, 71, 129, 75, 103, 69 | MS | 2 |
| 40 | 2-Monostearin | 17.31 | 1.46 | 1.51 | 103 | 103, 218, 217, 191, 203, 204, 219, 105 | MS | 2 |
| 41 | Cholesteryl propionate | 18.56 | 1.63 | 1.45 | 81 | 81, 95, 57, 105, 91, 107, 145, 93 | MS | 0 |
|  | *Indoles and derivatives* |  |  |  |  |  |  |  |
| 42 | 5-Hydroxyindole | 11.34 | 2.13 | 2.06 | 277 | 277, 278, 163, 276, 279, 205, 262, 202 | MS | 2 |
| 43 | 3-Indolepropionic acid | 13.87 | 1.34 | 1.62 | 202 | 202, 203, 333, 200, 334, 116, 215, 216 | MS | 2 |
|  | *Etcs.* |  |  |  |  |  |  |  |
| 44 | Ethyl dithioacetate | 4.13 | 2.13 | 1.56 | 120 | 59, 120, 75, 92, 58, 76, 61, 57 | MS | 0 |
| 45 | Ethyl isopropyl disulfide | 5.17 | 2.88 | 2.34 | 136 | 136, 66, 64, 59, 94, 61, 93, 60 | MS | 0 |
| 46 | Propane | 5.69 | 1.87 | 2.07 | 73 | 131, 75, 74, 132, 133, 148, 117, 59 | MS | 2 |
| 47 | Hydroxylamine | 5.97 | 1.86 | 1.37 | 73 | 133, 146, 119, 59, 249, 86, 72, 74 | MS | 3 |
| 48 | Diethyl trisulfide | 6.33 | 2.92 | 2.18 | 61 | 61, 154, 93, 64, 59, 89, 62, 91 | MS | 0 |
| 49 | Dodecane | 6.80 | 1.63 | 1.31 | 57 | 57, 71, 85, 56, 55, 84, 58, 69 | MS | 0 |
| 50 | Propargylamine | 7.71 | 2.25 | 1.65 | 199 | 86, 184, 199, 140, 59, 154, 81, 183 | MS | 2 |
| 51 | Catechol | 8.05 | 0.28 | 2.08 | 73 | 87, 254, 74, 59, 239, 75, 255, 151 | MS | 2 |
| 52 | 2-Nitrophenol | 8.81 | 1.65 | 1.28 | 196 | 196, 151, 74, 197, 104, 136, 57, 78 | MS | 1 |
| 53 | Pyrophosphoric acid | 11.00 | 0.79 | 1.32 | 451 | 451, 452, 299, 453, 207, 466, 77, 193 | MS | 4 |
| 54 | Glycerophosphoric acid | 11.69 | 1.60 | 1.47 | 299 | 299, 357, 101, 59, 75, 103, 129, 211 | MS | 4 |
| 55 | Bisphenol A | 14.66 | 0.66 | 1.83 | 357 | 357, 207, 358, 359, 372, 191, 128, 171 | MS | 2 |
|  | *N.I.D.^h^* |  |  |  |  |  |  |  |
|  | N.I.D. 1 | 7.04 | 2.91 | 2.21 | 120 | 120, 91, 148, 59, 89, 119, 209, 103 |  |  |
|  | N.I.D. 2 | 11.58 | 2.11 | 2.79 | 117 | 117, 75, 160, 118, 129, 103, 277, 74 |  |  |
|  | N.I.D. 3 | 14.43 | 2.86 | 2.35 | 130 | 130, 131, 159, 103, 276, 146, 132, 158 |  |  |

^a^Identified compounds based on the VIP 1 or 2 value (> 1.0) and *p* (< 0.05) from the PLS-DA model in Figure 3c; ^b^RT, retention time; ^c^VIP value based on PLS1; ^d^VIP value based on PLS2; ^e^Mass spectrum consistent with that of the standard compounds; ^f^Mass spectrum compared with the National Institute of Standards and Technology (NIST) database; ^g^TMS, Trimethylsilyl; ^h^N.I.D., Non-identified

**Supplementary Table 4.** Differential metabolites in plasma identified by UHPLC-Orbitrap-MS/MS.

| **No.** | **Tentative identification^a^** | **RT^b^ (min)** | **VIP 1^c^** | **VIP 2^d^** | **Precursor ion (m/z)** | **MS fragments^e^ (m/z)** | **Adduct** | **Exact mass** | **Formula** | **Error (ppm)** |
| --- | --- | --- | --- | --- | --- | --- | --- | --- | --- | --- |
|  | *Amino acids and derivatives* |  |  |  |  |  |  |  |  |  |
| 1 | Valine | 0.78 | 0.53 | 1.25 | 118.086 | (+) 72.080, 55.054, 59.073 | [M+H]+ | 117.079 | C5H11NO2 | -2.16 |
| 2 | Tyrosine | 1.12 | 1.24 | 1.21 | 182.081 | (+) 136.075, 162.054, 123.044 | [M+H]+ | 181.074 | C9H11NO3 | -0.57 |
| 3 | Phenylalanine | 1.12 | 1.40 | 0.95 | 166.086 | (+) 120.080, 131.048, 121.083 | [M+H]+ | 165.079 | C9H11NO2 | -2.74 |
| 4 | Leucine | 1.12 | 0.26 | 1.25 | 132.102 | (+) 86.096, 87.099, 69.069 | [M+H]+ | 131.094 | C6H13NO2 | -2.31 |
| 5 | Methionine* | 1.12 | 0.74 | 2.04 | 150.058 | (+) 104.053, 133.032, 56.049 | [M+H]+ | 149.051 | C5H11NO2S | -1.07 |
| 6 | 7-Keto-8-aminopelargonic acid | 5.91 | 0.10 | 1.14 | 188.128 | (+) 170.117, 152.106, 125.095 | [M+H]+ | 187.120 | C9H17O3N | -2.76 |
| 7 | N-Acetyltryptophan | 6.58 | 0.38 | 1.32 | 245.093 | (-) 203.084, 74.025, 116.035 | [M-H]- | 246.100 | C13H14N2O3 | -0.68 |
|  | *Bile acids and derivatives* |  |  |  |  |  |  |  |  |  |
| 8 | Tauroursocholic acid (TUCA) | 7.13 | 1.84 | 1.29 | 516.298 | (+) 462.266, 337.251, 126.021 | [M+H]+ | 515.290 | C26H45NO7S | -1.84 |
| 9 | Taurocholic acid (TCA)* | 7.84 | 2.08 | 1.40 | 538.280 | (+) 148.004, 484.248, 502.259 | [M+Na]+ | 515.291 | C26H45NO7S | -0.18 |
| 10 | Taurodeoxycholic acid (TDCA) | 8.78 | 1.81 | 1.22 | 500.303 | (+) 464.282, 339.268, 126.022 | [M+H]+ | 499.296 | C26H45NO6S | -1.47 |
|  | *Indoles and derivatives* |  |  |  |  |  |  |  |  |  |
| 11 | Indoline | 1.12 | 1.74 | 1.17 | 120.081 | (+) 120.081, 121.084, 103.054 | [M+H]+ | 119.073 | C8H9N | -2.30 |
| 12 | Indolepropionic acid* | 7.74 | 1.52 | 1.68 | 190.086 | (+) 130.065, 172.075, 167.977 | [M+H]+ | 189.079 | C11H11NO2 | 0.00 |
|  | *Carnitines and derivatives* |  |  |  |  |  |  |  |  |  |
| 13 | Carnitine* | 0.77 | 2.23 | 1.54 | 162.112 | (+) 103.039, 60.081, 163.115 | [M+H]+ | 161.105 | C7H15NO3 | 0.00 |
| 14 | Acetylcarnitine | 1.12 | 0.52 | 1.12 | 204.123 | (+) 85.028, 145.049, 60.081 | [M+H]+ | 203.115 | C9H17NO4 | -2.62 |
| 15 | Oleoylcarnitine | 10.48 | 1.08 | 0.97 | 426.357 | (+) 85.028, 60.081, 367.284 | [M+H]+ | 425.349 | C25H47NO4 | -2.78 |
|  | *Lipids and derivatives* |  |  |  |  |  |  |  |  |  |
| 16 | Lauric diethanolamide | 8.67 | 0.39 | 1.48 | 288.253 | (+) 217.195, 235.205, 95.085 | [M+H]+ | 287.246 | C16H33NO3 | -2.85 |
| 17 | Oleamide | 13.07 | 1.06 | 0.77 | 563.549 | (+) 247.241, 265.252, 97.101 | [2M+H]+ | 281.271 | C18H35ON | -3.08 |
| 18 | LPE(20:4) | 10.26 | 1.19 | 1.02 | 500.278 | (-) 303.233, 259.243, 196.038 | [M-H]- | 501.285 | C25H44NO7P | -0.52 |
| 19 | LysoPC(15:0) | 10.28 | 1.68 | 1.17 | 482.323 | (+) 184.073, 104.107, 86.096 | [M+H]+ | 481.316 | C23H48NO7P | -2.52 |
| 20 | LysoPC(22:6)* | 10.40 | 2.02 | 1.37 | 568.340 | (+) 184.073, 104.107, 86.096 | [M+H]+ | 567.333 | C30H50NO7P | 0.96 |
| 21 | LysoPC(18:2) | 10.43 | 1.42 | 1.23 | 520.338 | (+) 184.073, 104.107, 520.338 | [M+H]+ | 519.331 | C26H50NO7P | -2.63 |
| 22 | LysoPE(16:0) | 10.73 | 1.53 | 1.03 | 454.292 | (+) 313.273, 314.277 62.060 | [M+H]+ | 453.285 | C21H44NO7P | -2.02 |
| 23 | LysoPC(20:3) | 10.77 | 1.15 | 0.92 | 568.336 | (+) 184.073, 104.107, 86.096 | [M+Na]+ | 545.349 | C28H52NO7P | -0.81 |
| 24 | LysoPC(16:0) | 10.78 | 1.63 | 1.17 | 496.339 | (+) 184.073, 104.107, 478.328 | [M+H]+ | 495.331 | C24H50NO7P | -2.15 |
| 25 | LysoPE(18:1) | 11.02 | 1.14 | 0.84 | 480.307 | (+) 339.289, 340.292, 308.294 | [M+H]+ | 479.300 | C23H46O7NP | -2.22 |
| 26 | LPC O-16:0 | 11.05 | 1.14 | 0.82 | 482.361 | (+) 104.107, 184.073, 86.096 | [M+H]+ | 481.353 | C24H52NO6P | 0.00 |
| 27 | LysoPC(18:1) | 11.06 | 1.38 | 1.03 | 522.354 | (+) 184.073, 104.107, 504.344 | [M+H]+ | 521.347 | C26H52NO7P | -2.52 |
| 28 | LysoPC(P-16:0/0:0) | 11.08 | 1.09 | 0.74 | 480.345 | (+) 104.107, 184.073, 240.099 | [M+H]+ | 479.338 | C24H50NO6P | 0.00 |
| 29 | LysoPC(17:0) | 11.30 | 1.81 | 1.30 | 510.354 | (+) 184.073, 104.107, 509.538 | [M+H]+ | 509.347 | C25H52NO7P | -1.99 |
| 30 | LPI(18:2)* | 11.75 | 0.09 | 1.52 | 595.289 | (-) 279.233, 152.996, 241.012 | [M-H]- | 596.296 | C27H49O12P | 0.19 |
| 31 | LPE 18:0 | 11.80 | 1.17 | 0.79 | 482.323 | (+) 481.516, 314.304, 62.0599 | [M+H]+ | 481.316 | C23H48NO7P | -2.31 |
| 32 | LPI(20:4)* | 11.81 | 0.78 | 1.54 | 619.289 | (-) 303.233, 152.996, 241.012 | [M-H]- | 620.296 | C29H49O12P | 0.18 |
| 33 | LysoPC(18:0) | 11.86 | 1.71 | 1.22 | 524.370 | (+) 184.073, 104.107, 506.360 | [M+H]+ | 523.363 | C26H54NO7P | -2.41 |
| 34 | Lipid 1* | 9.72 | 0.44 | 1.64 | 338.266 | (+) 339.269, 337.253, 83.085 | [M+Na]+ | 315.276 |  |  |
| 35 | Lipid 2 | 10.09 | 1.38 | 0.93 | 516.304 | (+) 457.232, 104.107, 146.982 | [M+Na]+ | 493.316 |  |  |
| 36 | Lipid 3* | 10.40 | 1.98 | 1.34 | 590.320 | (+) 531.247, 104.107, 146.982 | [M+Na]+ | 567.331 |  |  |
| 37 | Lipid 4* | 10.46 | 2.41 | 1.69 | 566.320 | (+) 507.247, 146.982, 104.107 | [M+Na]+ | 543.331 |  |  |
| 38 | Lipid 5 | 10.64 | 1.71 | 1.15 | 570.354 | (+) 184.073, 104.107, 552.344 | [M+H]+ | 569.347 |  |  |
|  | *Etcs.* |  |  |  |  |  |  |  |  |  |
| 39 | Uric acid | 0.81 | 1.01 | 0.72 | 167.021 | (-) 124.015, 123.009, 95.014 | [M-H]- | 168.028 | C5H4N4O3 | -0.38 |
| 40 | Corticosterone | 8.38 | 0.15 | 1.57 | 347.221 | (+) 329.211, 121.065, 311.200 | [M+H]+ | 346.214 | C21H30O4 | -1.11 |
|  | *N.I.D.^f^* |  |  |  |  |  |  |  |  |  |
|  | N.I.D. 1 | 0.67 | 1.78 | 1.26 | 214.917 | (+) 158.927, 186.922, 84.959 |  |  |  |  |
|  | N.I.D. 2 | 1.12 | 0.33 | 1.30 | 276.143 | (+) 230.138, 258.133, 86.096 |  |  |  |  |
|  | N.I.D. 3 | 1.22 | 1.28 | 0.88 | 245.099 | (+) 246.103, 221.930, 199.086 |  |  |  |  |
|  | N.I.D. 4 | 5.91 | 0.59 | 1.21 | 210.110 | (+) 193.122, 81.033, 211.113 |  |  |  |  |
|  | N.I.D. 5 | 5.91 | 0.76 | 1.16 | 233.185 | (+) 170.117, 210.950, 232.091 |  |  |  |  |
|  | N.I.D. 6 | 6.61 | 0.60 | 1.01 | 184.133 | (+) 121.101, 139.115, 138.127 |  |  |  |  |
|  | N.I.D. 7 | 6.61 | 0.27 | 1.18 | 224.125 | (+) 200.972, 89.060, 225.128 |  |  |  |  |
|  | N.I.D. 8 | 6.67 | 1.01 | 1.36 | 614.344 | (+) 983.504, 383.275, 955.509 |  |  |  |  |
|  | N.I.D. 9 | 7.03 | 1.06 | 0.82 | 621.392 | (+) 133.085, 177.112, 221.137 |  |  |  |  |
|  | N.I.D. 10 | 7.16 | 0.66 | 1.12 | 238.141 | (+) 131.085, 237.163, 132.088 |  |  |  |  |
|  | N.I.D. 11 | 7.43 | 0.25 | 1.06 | 311.232 | (+) 259.169, 241.158, 231.174 |  |  |  |  |
|  | N.I.D. 12 | 7.76 | 0.27 | 1.03 | 347.229 | (+) 348.214, 346.198, 275.200 |  |  |  |  |
|  | N.I.D. 13 | 8.53 | 0.49 | 1.07 | 375.261 | (+) 376.265, 277.216, 294.242 |  |  |  |  |
|  | N.I.D. 14 | 9.22 | 1.16 | 0.79 | 443.232 | (+) 444.235, 415.201, 416.205 |  |  |  |  |
|  | N.I.D. 15* | 9.32 | 1.59 | 1.87 | 387.179 | (+) 105.070, 106.073, 119.049 |  |  |  |  |
|  | N.I.D. 16 | 9.94 | 0.09 | 1.15 | 437.192 | (+) 438.196, 303.119, 210.185 |  |  |  |  |
|  | N.I.D. 17 | 10.09 | 1.45 | 1.03 | 688.344 | (+) 385.273, 86.060, 140.070 |  |  |  |  |
|  | N.I.D. 18 | 10.84 | 1.45 | 0.97 | 296.258 | (+) 279.231, 153.127, 81.070 |  |  |  |  |

^a^Identified compounds based on the VIP 1 or 2 value (> 1.0) from the PLS-DA model in Figure 3d; ^b^RT, retention time; ^c^VIP value based on PLS1; ^d^VIP value based on PLS2; ^e^Mass fragment patterns detected in positive and negative mode; ^f^N.I.D, Non-identified. (* *p* < 0.05)

**
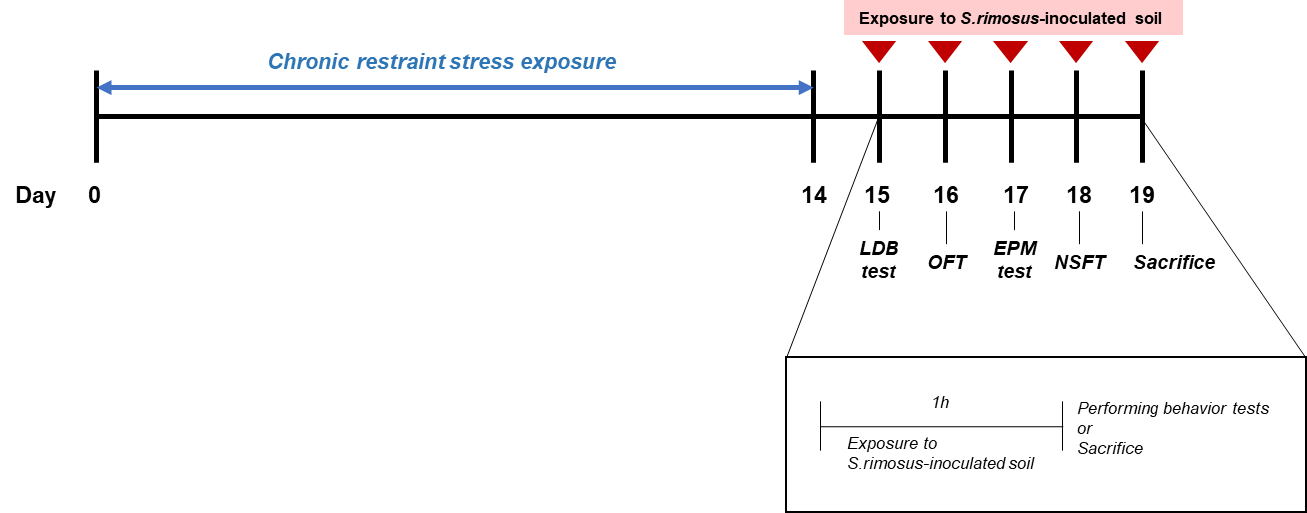
**

**Supplementary Figure S1. Experimental timeline**

Schematic representation of the experimental timeline. Mice were subjected to chronic restraint stress (CRS) for 6 h/day over 14 consecutive days (days 1–14). From day 15 to day 18, behavioral tests were conducted once per day in the following order: light–dark box (LDB), open field test (OFT), elevated plus maze (EPM), and novelty-suppressed feeding test (NSFT). On day 19, mice were exposed to *S. rimosus*–inoculated soil for 1 h and then sacrificed for tissue collection.


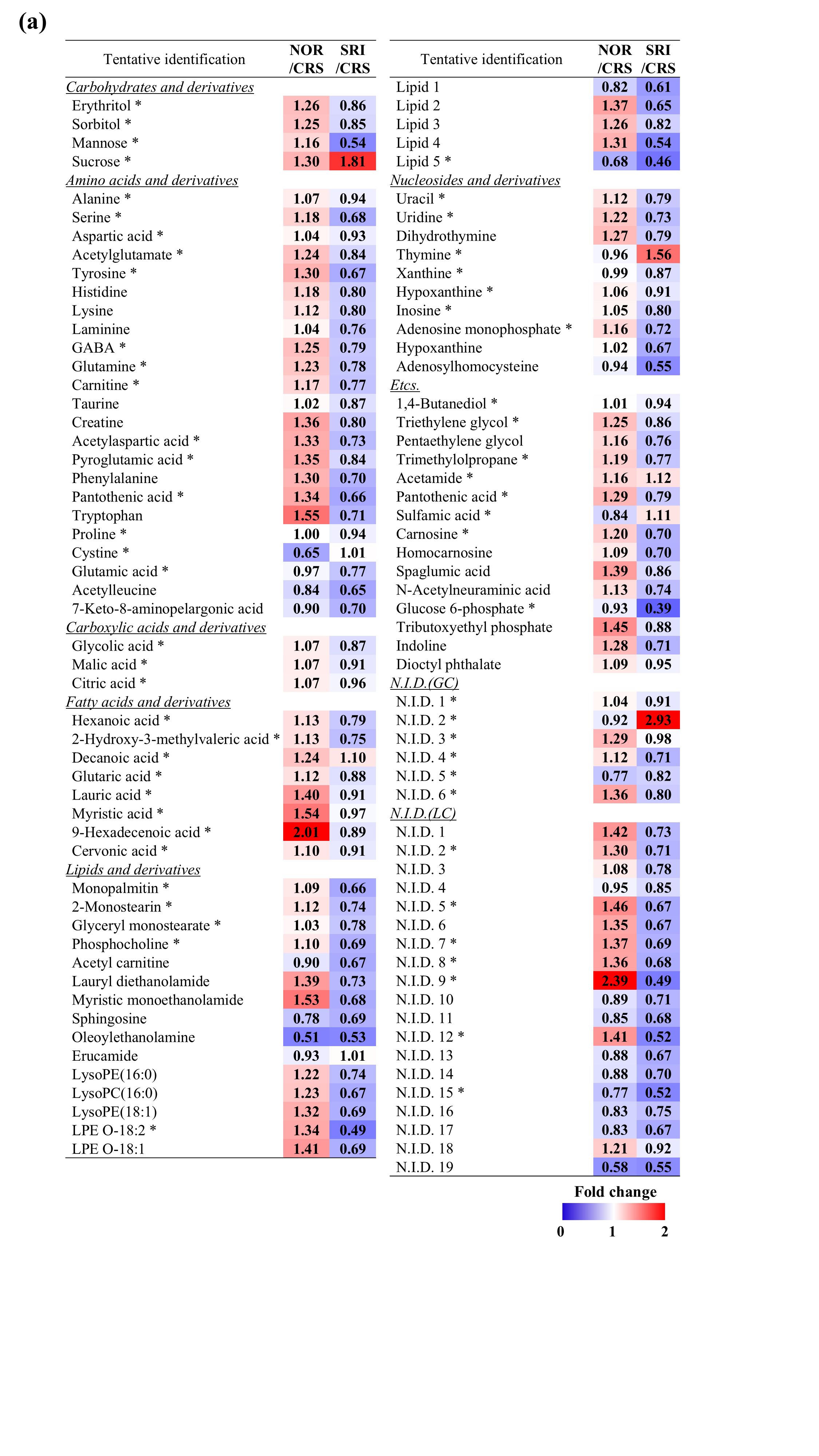


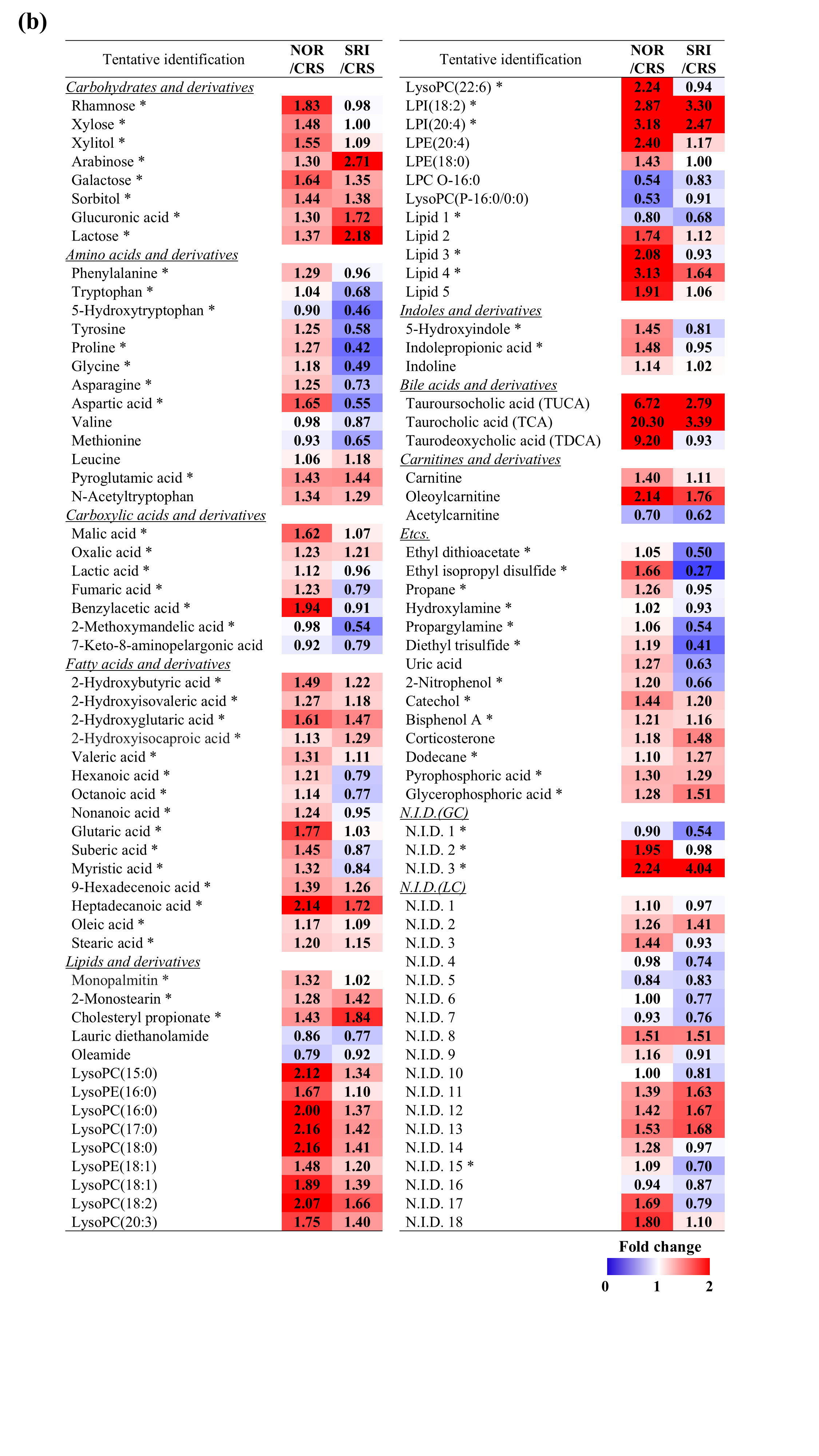
**Supplementary Figure S2.** Heatmap analysis of the relative abundance of differential metabolites identified by GC-TOF-MS and UHPLC-Orbitrap-MS/MS.

Relative abundance is shown compared to the CRS group. (a) Hippocampus; (b) Plasma. Statistically significant metabolites are indicated (* *p* < 0.05).


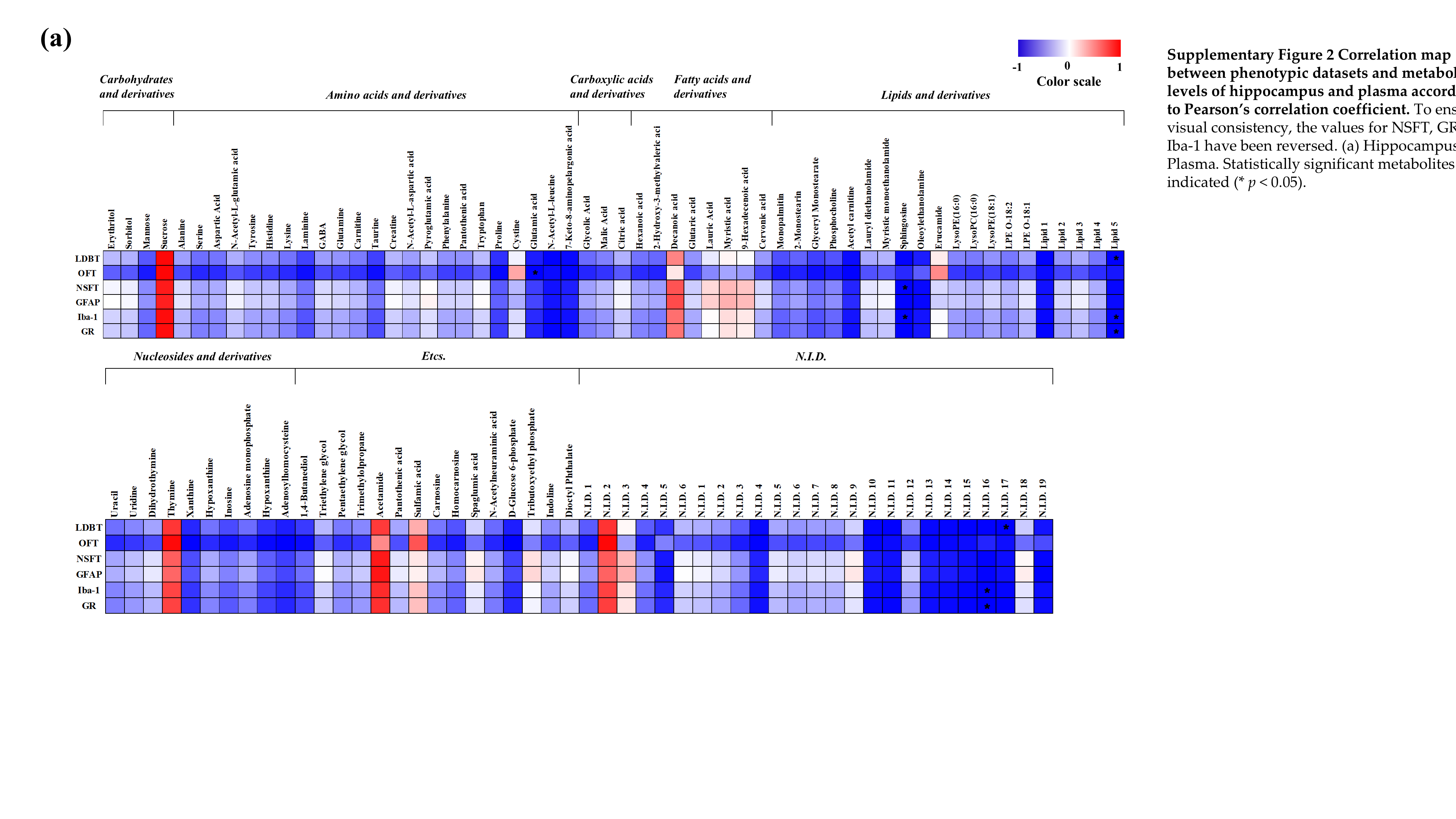

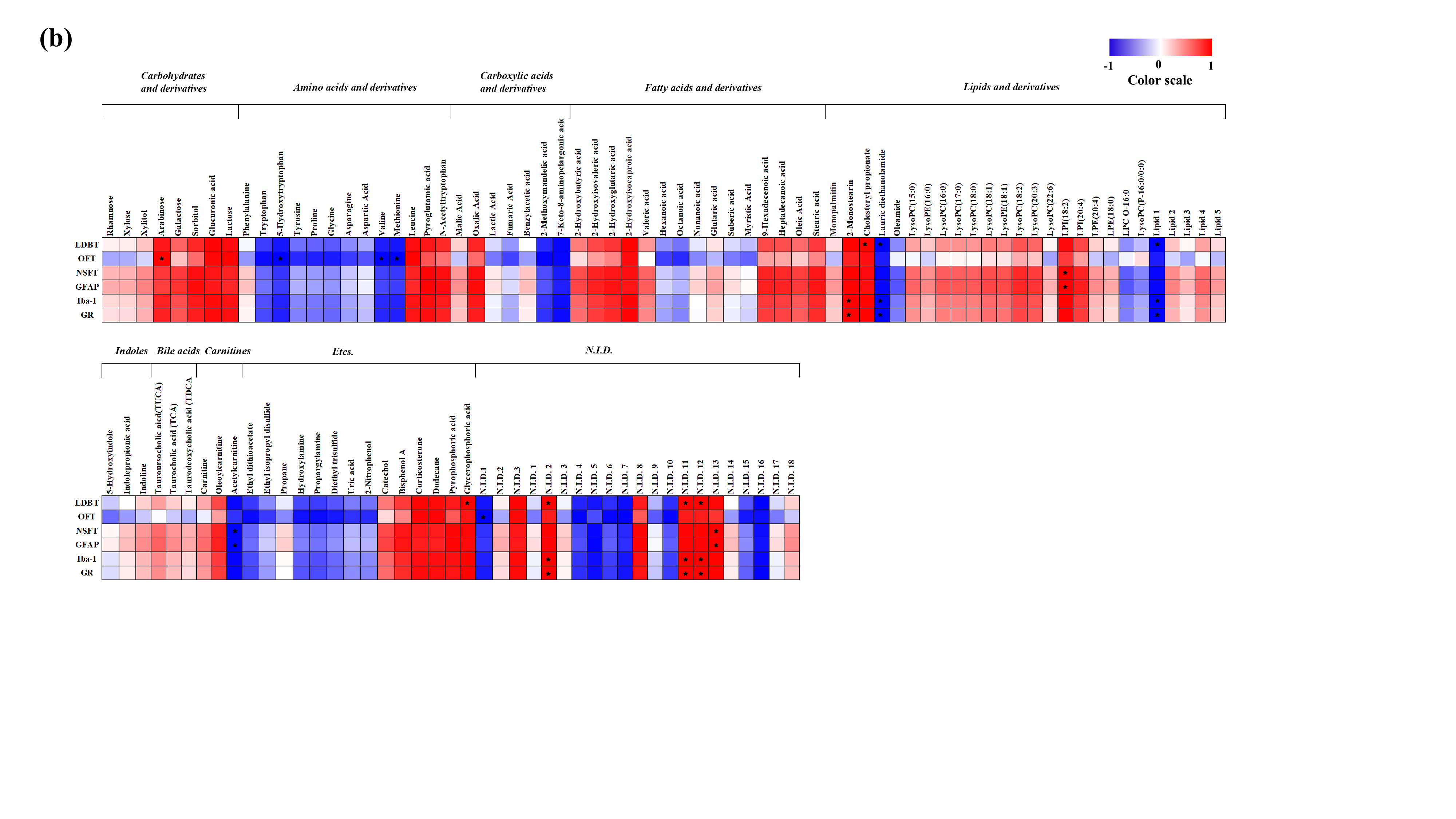
**Supplementary Figure S3.** Correlation map between phenotypic datasets and metabolite levels of hippocampus and plasma according to Pearson’s correlation coefficient.

To ensure visual consistency, the values for NSFT, GRAP, Iba-1 have been reversed. (a) Hippocampus; (b) Plasma. Statistically significant metabolites are indicated (* *p* < 0.05).
